# S-nitrosylation attenuates pregnane X receptor hyperactivity and acetaminophen-induced liver injury

**DOI:** 10.1172/jci.insight.172632

**Published:** 2024-01-23

**Authors:** Qi Cui, Tingting Jiang, Xinya Xie, Haodong Wang, Lei Qian, Yanyan Cheng, Qiang Li, Tingxu Lu, Qinyu Yao, Jia Liu, Baochang Lai, Chang Chen, Lei Xiao, Nanping Wang

**Affiliations:** 1 Advanced Institute for Medical Sciences, Dalian Medical University, Dalian, China.; 2 School of Basic Medical Sciences, Xi’an Jiaotong University, Xi’an, China.; 3 East China Normal University Health Science Center, Shanghai, China.; 4 School of Public Health, Xi’an Jiaotong University, Xi’an, China.; 5 National Laboratory of Biomacromolecules, Center for Excellence in Biomacromolecules, Institute of Biophysics, Chinese Academy of Sciences, Beijing, China.

**Keywords:** Cell Biology, Hepatology, Molecular biology, Nitric oxide

## Abstract

Drug-induced liver injury (DILI), especially acetaminophen overdose, is the leading cause of acute liver failure. Pregnane X receptor (PXR) is a nuclear receptor and the master regulator of drug metabolism. Aberrant activation of PXR plays a pathogenic role in the acetaminophen hepatotoxicity. Here, we aimed to examine the S-nitrosylation of PXR (SNO-PXR) in response to acetaminophen. We found that PXR was S-nitrosylated in hepatocytes and the mouse livers after exposure to acetaminophen or S-nitrosoglutathione (GSNO). Mass spectrometry and site-directed mutagenesis identified the cysteine 307 as the primary residue for S-nitrosylation (SNO) modification. In hepatocytes, SNO suppressed both agonist-induced (rifampicin and SR12813) and constitutively active PXR (VP-PXR, a human PXR fused to the minimal transactivator domain of the herpes virus transcription factor VP16) activations. Furthermore, in acetaminophen-overdosed mouse livers, PXR protein was decreased at the centrilobular regions overlapping with increased SNO. In *PXR^–/–^* mice, replenishing the livers with the SNO-deficient PXR significantly aggravated hepatic necrosis, increased HMGB1 release, and exacerbated liver injury and inflammation. Particularly, we demonstrated that S-nitrosoglutathione reductase (GSNOR) inhibitor N6022 promoted hepatoprotection by increasing the levels of SNO-PXR. In conclusion, PXR is posttranslationally modified by SNO in hepatocytes in response to acetaminophen. This modification mitigated the acetaminophen-induced PXR hyperactivity. It may serve as a target for therapeutical intervention.

## Abstract 2

<p>S-nitrosylation negatively regulates the activity of xenobiotic receptor PXR and mitigates drug-induced hepatotoxicity in a mouse model of acetaminophen overdose.</p>

## Introduction

The liver plays a critical role in the metabolism and clearance of drugs through the detoxification system, including phase I cytochrome P450 (CYP) enzymes, phase II–conjugating enzymes, and transporters (1). Overdose or accumulated exposure of drugs induces liver injury. Drug-induced liver injury (DILI) is a leading cause of acute liver failure (2). Acetaminophen (APAP) is a commonly used over-the-counter antipyretic and analgesic. APAP overdose is the leading cause of drug-induced acute liver failure (3, 4).

In adults receiving therapeutic dosages, around 63% of APAP is metabolized through glucuronidation, while approximately 34% is metabolized through sulfation (5). The remaining approximately 5% is oxidized by CYP enzymes, including CYP2E1, CYP1A2, and CYP3A4, forming the reactive intermediate N-acetyl-p-benzoquinone imine (NAPQI). NAPQI can be rapidly eliminated by binding with glutathione (GSH), forming its major nontoxic metabolites (APAP-sulfate, APAP-glucuronide [Glu], APAP-GSH, and APAP-cysteine [Cys]) (1, 6). However, when GSH levels are depleted, NAPQI accumulates in hepatocytes, causing mitochondrial oxidative stress, DNA damage, generation of reactive oxygen species (ROS), and ultimately hepatocyte necrosis and acute liver failure (7, 8).

Pregnane X receptor (PXR) is a member of the nuclear receptor superfamily and is primarily expressed in the liver and intestine (9). As a sensor for xenobiotics, including drugs and environmental chemicals, PXR can be activated by a variety of such structurally diverse ligands (10). PXR binds to the DNA *cis*-element to transactivate a plethora of its target genes involving the biotransformation and clearance of these chemicals (11). Induction of the phase I detoxification genes, such as *CYP3A4*, converts APAP to NAPQI and leads to hepatotoxicity (12). In mice, the PXR activator pregnenolone 16α-carbonitrile (PCN) exacerbates APAP-induced liver injury (AILI) in a PXR-dependent manner (13). APAP hepatotoxicity is reduced in *PXR^–/–^* mice (14). Rifampicin enhances hepatic toxicity in mice transgenic for the human *PXR* and *CYP3A4* (15). These results suggest that PXR plays a key role in the pathogenesis of AILI through drug-induced transcriptional activation. However, PXR posttranslational modifications and their regulatory roles in AILI remain to be explored.

Protein S-nitrosylation (SNO) is a redox-sensitive modification at cysteine residues by nitric oxide (NO) or NO-derived species (16). SNO regulates the substrate proteins by modifying their activity, stability, subcellular localization, or interaction with other molecules (17, 18). In the process of drug metabolism, a large amount of ROS and reactive nitrogen species were produced (19). S-nitrosol signaling is implicated in APAP-triggered liver damage in zebrafish and mice. The deficiency of S-nitrosoglutathione reductase (GSNOR), an enzyme responsible for denitrosylation, has a hepatoprotective effect (20). In addition, a redox proteomic analysis revealed that APAP induced protein SNO in 3-D cultured human hepatocyte lines (21). These results prompt us to investigate (a) whether PXR could be S-nitrosylated and, if so, (b) its regulatory roles in AILI.

In this study, we demonstrate that SNO posttranslationally modifies PXR activity and protein stability. Such a modification ameliorates APAP-induced hepatotoxicity by establishing an inhibitory mechanism for the PXR hyperactivity.

## Results

### PXR was S-nitrosylated in cultured hepatocytes and mouse livers.

**Figure 1:**
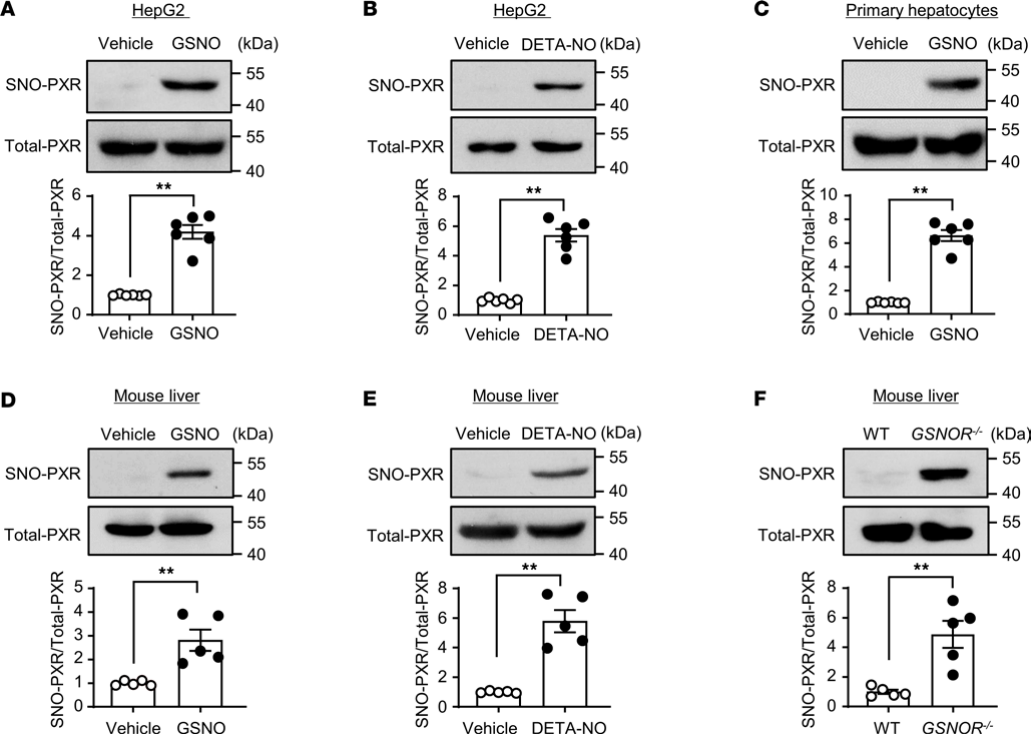
PXR was S-nitrosylated in cultured hepatocytes and mouse livers. (**A**) HepG2 cells were infected with Ad-PXR for 24 hours and exposed to GSNO (0.5 mM, 4 hours). Cell lysates were subjected to IBP to detect S-nitrosylation of PXR (SNO-PXR) level (*n* = 6). (**B**) HepG2 cells were exposed to DETA-NO (1 mM, 12 hours) (*n* = 6). (**C**) Mouse primary hepatocytes were exposed to GSNO (0.5 mM, 4 hours) (*n* = 6). (**D** and **E**) C57BL/6J mouse liver tissues were homogenized, and the lysates were treated with GSNO (0.5 mM, 1 hour) (**D**) or DETA-NO (1 mM, 1 hour) (**E**) and subjected to IBP (*n* = 5). (**F**) WT and *GSNOR*^–/–^ mouse liver tissues were homogenized, and the lysates were subjected to IBP (*n* = 5). All data were expressed as mean ± SEM. Statistical analysis was performed using 2-tailed Student’s *t* test; **P < 0.01.

To examine whether PXR protein can be modified by SNO, we infected HepG2 cells with adenovirus-expressing PXR (Ad-PXR) and, 24 hours later, exposed the cells to NO donors or vehicle control. S-nitrosylated PXR (SNO-PXR) was detected with an irreversible biotinylation procedure (IBP) followed by Western blotting. As shown in Figure 1A, PXR underwent SNO after the treatment with GSNO (0.5 mM, 4 hours), an amino acid transnitrosation donor. The PXR overexpression was validated (Supplemental Figure 1; supplemental material available online with this article; https://doi.org/10.1172/jci.insight.172632DS1). The specificity of IBP reaction was validated in the absence of ascorbate (Supplemental Figure 2). Another NO donor DETA-NONOate (1 mM, 12 hours) also elicited the SNO-PXR (Figure 1B). In addition, endogenous PXR was also modified by protein SNO in mouse primary hepatocytes (Figure 1C). SNO-PXR was further confirmed in mouse liver protein lysates after the treatment with GSNO or DETA-NONOate (Figure 1, D and E).

Since GSNOR reverses protein SNO (22), we compared the levels of SNO-PXR in the liver samples from *GSNOR*-KO (*GSNOR^–/–^*) mice and the WT littermates. As shown in Figure 1F, the levels of SNO-PXR were significantly elevated in livers from the *GSNOR^–/–^* mice. The *GSNOR^–/–^* and WT mice were genotyped by using PCR (Supplemental Figure 3A). The deficiency of GSNOR in KO mouse livers was confirmed by using Western blotting (Supplemental Figure 3B). Immunofluorescence staining for SNO-cysteine showed that global protein-SNO levels were increased in the livers of *GSNOR^–/–^* mice (Supplemental Figure 3C). Taken together, PXR was posttranslationally modified via protein SNO both in cultured hepatocytes and mouse livers.

### SNO suppressed the transactivational capacity of PXR.

**Figure 2:**
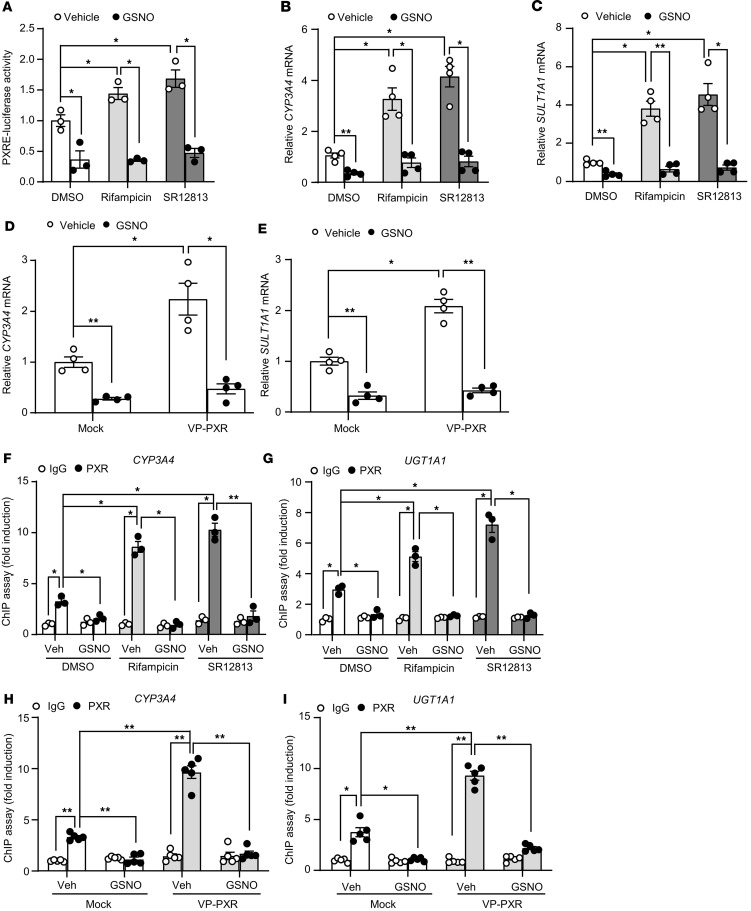
S-nitrosylation suppressed the transactivatioal capacity of PXR. (**A**) HepG2 cells were cotransfected with HA-PXR, PXRE-luciferase receptor, and β-gal plasmids. After 24-hour transfection, cells were pretreated with GSNO (0.5 mM, 4 hours) before the exposure to rifampicin (10 μM, 24 hours) or SR12813 (1 μM, 24 hours). The luciferase activities were measured and normalized to β-gal activity (*n* = 3). (**B** and **C**) After pretreatment with GSNO, HepG2 cells were stimulated with rifampicin or SR12813 for 24 hours. The mRNA levels of *CYP3A4* (**B**) and *SULT1A1* (**C**) were assessed by using qPCR (*n* = 4). (**D** and **E**) HepG2 cells were coinfected with Ad–VP-PXR together with Ad-tTA in the presence (Mock) or absence of Tc (1 μg/mL) for 24 hours and then exposed to GSNO (0.5 mM, 24 hours). The mRNA levels of *CYP3A4* (**D**) and *SULT1A1* (**E**) were assessed (*n* = 4). (**F** and **G**) After pretreatment with GSNO, HepG2 cells were stimulated with rifampicin or SR12813 for 24 hours. ChIP assays were performed with PXR antibody and primers flanking the PXRE motif in the *CYP3A4* (**F**) and *UGT1A1* (**G**) promoter region. The qPCR results were expressed as fold change compared with IgG control (*n* = 3). (**H** and **I**) HepG2 cells were coinfected with Ad–VP-PXR together with Ad-tTA in the presence (Mock) or absence of Tc (1 μg/mL) for 24 hours and then exposed to GSNO (0.5 mM, 24 hours). ChIP assays were performed with PXR antibody and primers flanking the PXRE motif in the *CYP3A4* (**H**) and *UGT1A1* (**I**) promoter region (*n* = 5). Data were expressed as mean ± SEM. Statistical analysis was performed using 1-way ANOVA followed by Tukey’s multiple-comparison test; *P < 0.05, **P < 0.01.

Since PXR is a ligand-activated transcription factor, we first investigated whether SNO affects the ligand-induced transactivational capacity of PXR. HepG2 cells were cotransfected with the *PXR* plasmid and the PXR responsive element–luciferase (PXRE-luciferase) receptor plasmid and, 24 hours later, treated with GSNO or control for 4 hours before the exposure to PXR agonist rifampicin (10 μM) or SR12813 (1 μM) for 24 hours. The luciferase assays showed that GSNO significantly decreased the PXR reporter activity under basal condition or upon the agonist stimulation (Figure 2A). Next, we examined the effects of SNO-PXR on the transactivation of its endogenous target gene *CYP3A4* and *SULT1A1*. GSNO treatment significantly attenuated the mRNA levels of *CYP3A4* and *SULT1A1* genes induced by rifampicin or SR12813 as assessed by using quantitative PCR (qPCR) (Figure 2, B and C). In addition, GSNO prevented the induction of *CYP3A4* and *SULT1A1* genes transactivated by a constitutively active PXR (VP-PXR, a human PXR fused to the minimal transactivator domain of the herpes virus transcription factor VP16) (Figure 2, D and E). The efficiency of PXR overexpression was validated (Supplemental Figure 4).

We further examined the effects of the SNO on the DNA binding of PXR to the 5′-regulatory regions within its target genes. As shown in Figure 2, F and G, ChIP assays revealed that GSNO treatment significantly reduced basal and the rifampicin- or SR12813-stimulated PXR binding onto the specific regions harboring the cognate DNA elements within the *CYP3A4* and *UGT1A1* genes. The DNA binding of VP-PXR to *CYP3A4* and *UGT1A1* genes was also largely abrogated after GSNO treatment (Figure 2, H and I). Taken together, these results indicate that the SNO negatively regulated the transactivational capacity of PXR by decreasing its binding to the target genes.

### SNO destabilized PXR protein via proteasome-dependent degradation.

**Figure 3:**
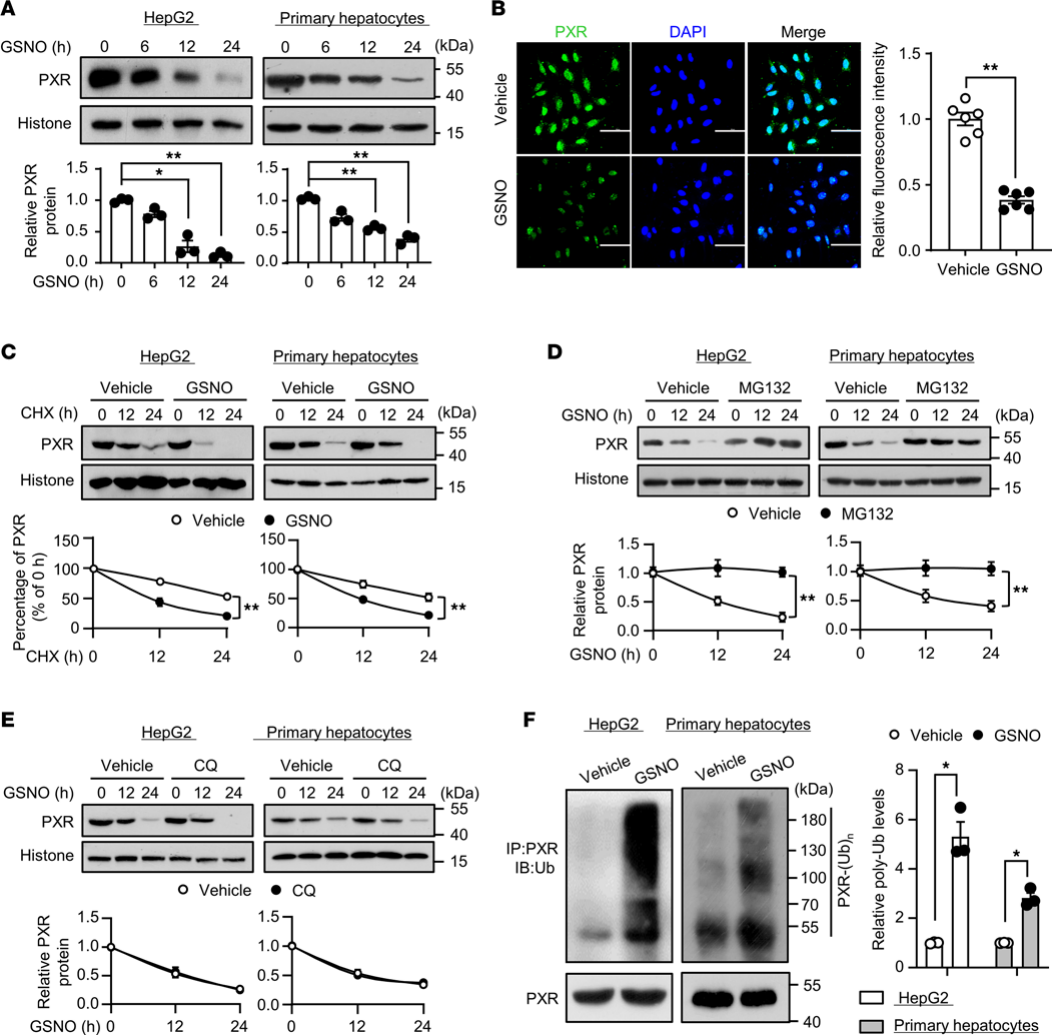
S-nitrosylation destabilized PXR protein via proteasome-dependent degradation. (**A**) HepG2 cells were infected with Ad-PXR for 24 hours, or mouse primary hepatocytes were exposed to GSNO (0.5 mM) for indicated times. Nuclear PXR protein level was detected by using Western blotting (*n* = 3). (**B**) HepG2 cells were infected with Ad-PXR for 24 hours before being exposed to GSNO (0.5 mM, 12 hours) and were then subjected to immunofluorescence staining for PXR. Nuclei were counterstained with DAPI. Graph shows the mean fluorescence intensity (*n* = 6). Scale bars: 100 μm. (**C**) HepG2 cells or mouse primary hepatocytes were pretreated with CHX (30 μg/mL, 1 hour) before the exposure to GSNO for indicated times. Nuclear proteins were extracted and analyzed (*n* = 3). (**D** and **E**) HepG2 cells or mouse primary hepatocytes were pretreated with MG132 (10 μM, 1 hour) (**D**) or CQ (20 μM, 1 hour) (**E**) before the exposure to GSNO for indicated times. Nuclear proteins were extracted and analyzed (*n* = 3). (**F**) HepG2 cells or mouse primary hepatocytes were treated with GSNO (0.5 mM, 12 hours) and MG132 (10 μM, 6 hours) before harvesting. PXR was immunoprecipitated using anti-PXR antibody. The immunoprecipitates (IP) and whole-cell extracts were analyzed by Western blotting using an anti-Ub and anti-PXR antibody, respectively (*n* = 3). All data were expressed as mean ± SEM. Statistical analysis was performed using 1-way ANOVA followed by Tukey’s multiple-comparison test (**A**), 2-tailed Student’s *t* test (**B** and **F**), or multiple-comparison 2-way ANOVA with Bonferroni’s post hoc test (**C**, **D**, and **E**); *P < 0.05, **P < 0.01.

One of the important functional abilities of the protein SNO is the ability to change the stability of the modified substrates via ubiquitination and the ensuing degradation (23). Thus, we determined whether SNO affected the protein stability of PXR. HepG2 cells were infected with Ad-PXR, and 24 hours later, were exposed to GSNO. The PXR protein level was significantly decreased after GSNO treatment. PXR protein levels were also decreased in GSNO-treated mouse primary hepatocytes (Figure 3A). Immunofluorescence staining confirmed the decrease of PXR in HepG2 cells (Figure 3B). However, GSNO did not affect the mRNA levels of PXR (Supplemental Figure 5).

To examine the effect of SNO on the protein stability of PXR, we treated the HepG2 cells or mouse primary hepatocytes with cycloheximide (CHX) to inhibit protein de novo synthesis. As shown in Figure 3C, the half-life of PXR protein was decreased in the cells treated with GSNO. In addition, the proteasome inhibitor MG132, but not the lysosome inhibitor chloroquine (CQ), prevented the GSNO-induced decrease in PXR protein levels (Figure 3, D and E). We further showed that GSNO treatment triggered PXR ubiquitination (Figure 3F). These findings suggest that SNO decreased PXR protein stability through protein ubiquitination and proteasome-dependent degradation.

### Identification of the S-nitrosylated cysteine residues in the human PXR.

Next, we generated a histidine tag (6×His)-PXR recombinant protein and exposed it to GSNO. Then, the S-nitrosylated cysteines were labeled with biotin-maleimide and subjected to liquid chromatography with tandem mass spectrometry (LC-MS/MS) to identify the potential cysteine residues undergoing SNO. As shown in Supplemental Figures 6 and 7, the cysteine residues at 182 and 307 were found to be S-nitrosylated.

**Figure 4:**
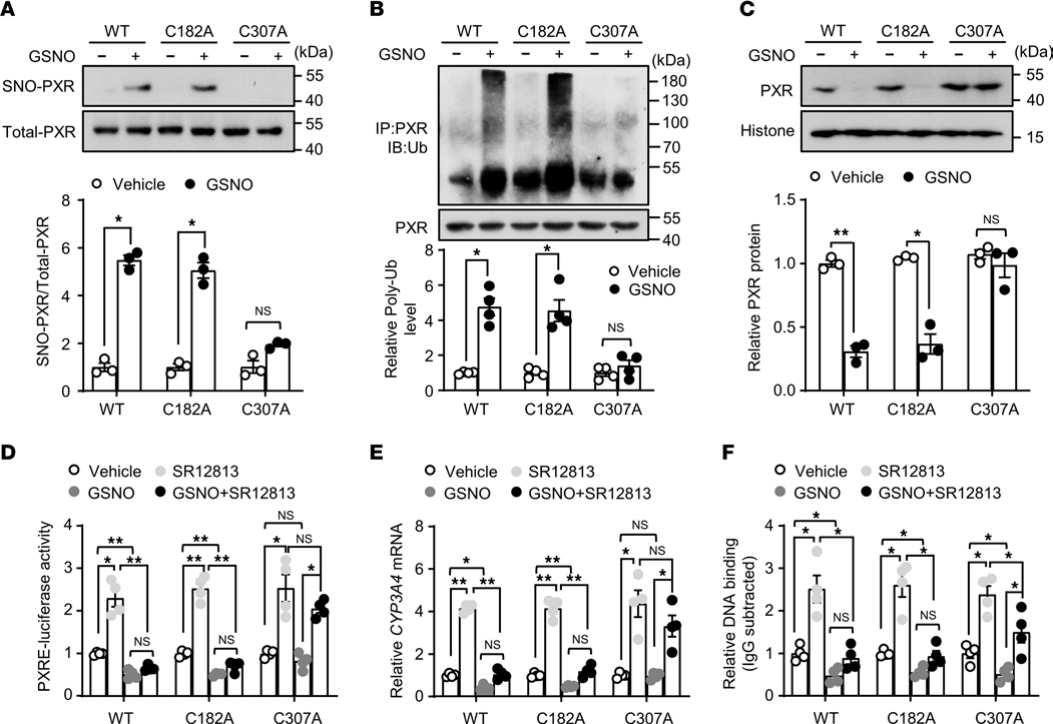
Identification of the S-nitrosylated cysteine residues in the human PXR. (**A**) HEK293 cells were transfected with PXR WT or mutants (C182A, C307A) plasmids (24 hours) before the exposure to GSNO (0.5 mM, 4 hours). Cell lysates were subjected to IBP to detect S-nitrosylated PXR level (*n* = 3). (**B**) Transfected cells were treated with GSNO (0.5 mM, 12 hours) and MG132 for 6 hours before harvesting. PXR was immunoprecipitated using anti-PXR antibody. The IP and whole-cell extracts were analyzed by Western blotting using an anti-Ub and anti-PXR antibody, respectively (*n* = 4). (**C**) HEK293 cells were transfected with WT, C182A, or C307A plasmids for 24 hours and then treated with GSNO (0.5 mM, 12 hours). Nuclear PXR protein level was detected by using Western blotting (*n* = 3). (**D**) PXR WT, C182A, or C307A was cotransfected with PXRE-luciferase receptor into HEK293 cells. After 24 hours of transfection, cells were pretreated with GSNO (0.5 mM, 4 hours) before the exposure to SR12813 (1 μM, 24 hours). The luciferase activities were measured and normalized to β-gal activity (*n* = 4). (**E**) HEK293 cells were transfected with PXR WT, C182A, or C307A for 24 hours. After pretreatment with GSNO (0.5 mM, 4 hours), the cells were stimulated with SR12813 (1 μM, 24 hours). The mRNA level of *CYP3A4* was assessed (*n* = 4). (**F**) ChIP assays were performed (*n* = 4). All data were expressed as mean ± SEM. Statistical analysis was performed using 1-way ANOVA followed by Tukey’s multiple-comparison test; *P < 0.05, **P < 0.01.

To verify the sites of SNO, we replaced the corresponding cysteines with alanine residues (C182A, C307A) in the PXR by using site-directed mutagenesis. HEK293 cells were transfected with expression vectors encoding WT or mutant PXR. HEK293 cells were used because they have better transfection efficiency with plasmids and have little expression of endogenous PXR (24). The lack of PXR expression in HEK293 cells was confirmed (Supplemental Figure 8). As shown in Figure 4A, the mutation at PXR Cys307 but not Cys182 abolished the SNO-PXR induced by GSNO, suggesting that the cysteine residue at 307 was a key moiety for intracellular SNO. Consistently, the SNO at Cys307 was predominately responsible for the ubiquitination and degradation of PXR (Figure 4, B and C). Functionally, disrupting SNO at Cys307 but not Cys182 largely restored the transactivational activity on the luciferase reporter gene and the expression of PXR target gene *CYP3A4* (Figure 4, D and E). As shown in Figure 4F, GSNO blunted the SR12813-induced binding of PXR-WT onto the DNA segment of *CYP3A4* gene. However, the mutation on C307 (C307A) but not on C182 restored the SR12813-induced DNA binding upon GSNO treatment. Taken together, these results suggest that SNO modified PXR predominantly at Cys307.

### SNO-PXR was triggered in response to APAP-induced hepatotoxicity.

**Figure 5:**
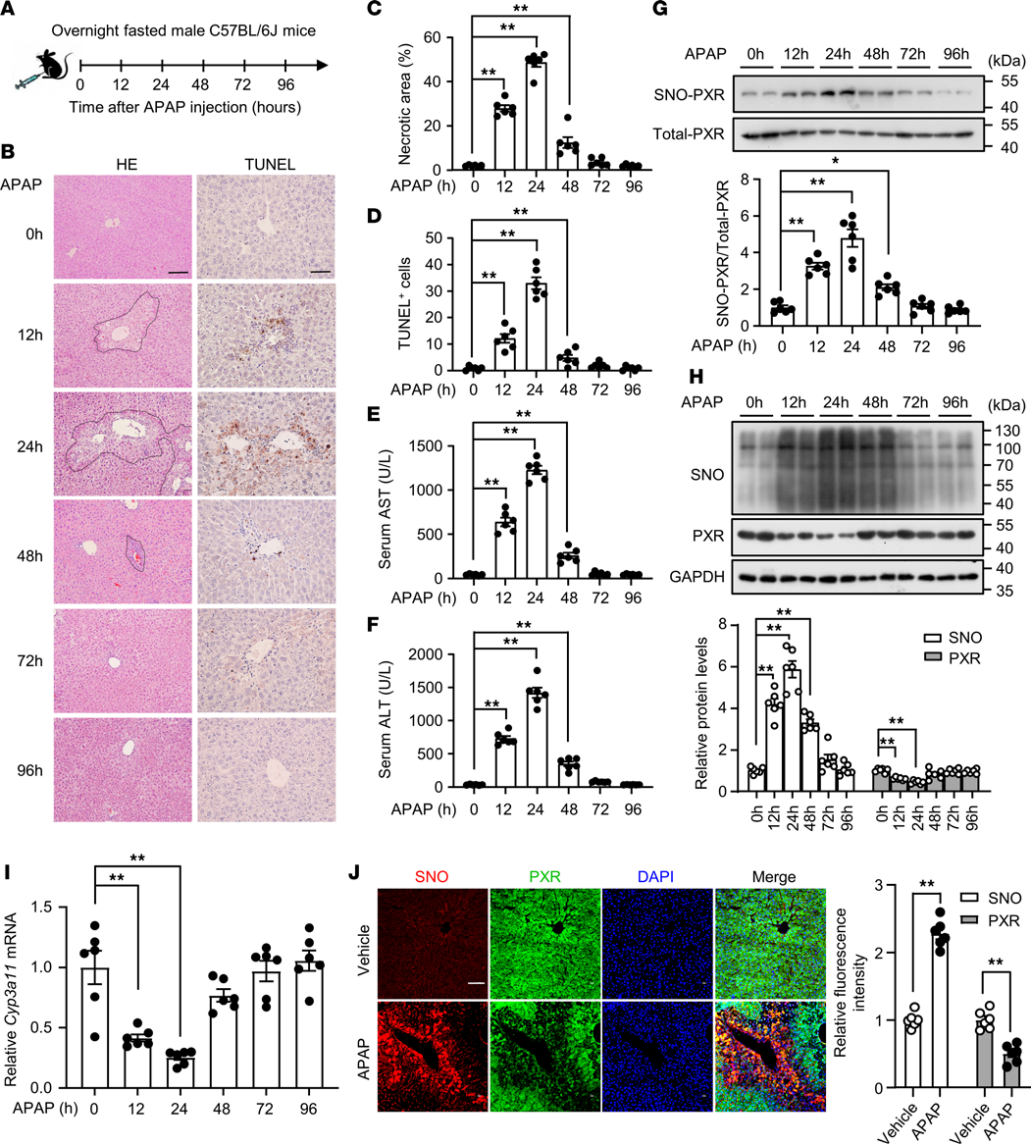
PXR S-nitrosylation was triggered in response to acetaminophen-induced hepatotoxicity. (**A**) A schematic of the experimental design. C57BL/6J mice at 8–10 weeks of age were i.p. injected with acetaminophen (APAP, 300 mg/kg) and sampled 12, 24, 48, 72, and 96 hours later. (**B**) Mouse liver tissues were fixed with 4%PFA and liver sections were used for H&E (scale bars: 100 μm) and TUNEL (scale bars: 50 μm) staining (*n* = 6). (**C**) Necrotic areas were quantified by using the ImageJ software. (**D**) TUNEL^+^ cells were counted in 6 randomly selected high-power fields per liver section. (**E** and **F**) Serum levels of AST (**E**) and ALT (**F**) in mice (*n* = 6). (**G**) Levels of S-nitrosylated PXR in the liver tissues (*n* = 6). (**H**) The total SNO-cysteine (SNO) and PXR protein levels in liver tissues (*n* = 6). (**I**) The mRNA level of *Cyp3a11* was assessed (*n* = 6). (**J**) Mouse liver sections were subjected to immunofluorescence double-stained with primary antibodies against PXR and SNO, and followed by the detection with Alexa Fluor 488–conjugated (green) and Alexa Fluor 555–conjugated (red) secondary antibodies. The cell nuclei were counterstained with DAPI. Scale bars: 100 μm. (*n* = 6). All data were expressed as mean ± SEM. Statistical analysis was performed using 1-way ANOVA followed by Tukey’s multiple-comparison test (**C**–**I**) or 2-tailed Student’s *t* test (**J**); *P < 0.05, **P < 0.01.

To explore a physiopathlogical role of SNO-PXR in the liver, we established a DILI mouse model with APAP overdose. C57BL/6J mice were treated with a single overdose of APAP (300 mg/kg, i.p. injection) after a 12-hour fasting. The histopathological examination revealed that APAP-treated mice exhibited centrilobular hepatocyte necrosis coincident with increased positivity of TUNEL (Figure 5, A–D). The circulating levels of aspartate aminotransaminase (AST) and alanine aminotransferase (ALT) were increased (Figure 5, E and F). Importantly, SNO of PXR was significantly increased in the livers of APAP-overdosed mice, starting at 12 hours with a peak at 24 hours after the APAP (Figure 5G). In contrast, PXR protein levels were concomitantly decreased in the livers of APAP-treated mice with significantly reduced mRNA levels of the PXR target gene *Cyp3a11* (Figure 5, H and I). Immunofluorescence staining with an antibody against SNO-cysteine revealed that APAP caused a robust accumulation of SNO products predominately at the centrilobular zone, where the APAP-induced hepatic necrosis occurred. Coincidently, the PXR protein levels in these regions exhibited a significant decrease (Figure 5J). These results suggested that SNO-PXR was implicated in APAP-induced hepatotoxicity.

Induction of inducible NO synthase (iNOS) accounts for a major source of NO in many types of tissues (25). Upregulation of iNOS, the predominant NOS isoform expressed in hepatocytes, has been observed in multiple models of liver injury (26–28). Therefore, we observed the changes of iNOS expression in the livers of APAP-treated mice. As shown in Supplemental Figure 9A, the protein level of iNOS, but not endothelial NOS (eNOS), was significantly increased. In addition, siRNA-mediated knockdown of *Inos* reduced SNO-PXR in mouse primary hepatocytes (Supplemental Figure 9B).

### Liver replenishment with the SNO-mutant PXR exacerbated APAP-induced hepatotoxicity in PXR-null mice.

To further define the pathological role of SNO-PXR in APAP-induced hepatotoxicity, we generated *PXR^–/–^* mice by using a CRISPR/Cas9 gene-editing technique (Supplemental Figure 10A). PXR deficiency was confirmed with PCR genotyping of genomic DNA and Western blotting (Supplemental Figure 10, B and C). We also constructed adenoviruses carrying WT human PXR (Ad–PXR-WT) and the SNO-mutant PXR (Ad–PXR-C307A). The infection efficiency of adenovirus was also determined. We administered tail vein injections of Ad–PXR-WT and control Ad-EGFP into WT and the *PXR^–/–^* mice. Western blotting showed that adenovirus-mediated delivery of WT or mutant PXR reconstructed PXR expressions in the *PXR^–/–^* mice. Because the PXR-WT and the PXR-C307A, respectively, carried EGFP and mCherry, their expression levels were readily detectable in the livers of the *PXR^–/–^* mice under fluorescence microscopy (Supplemental Figure 11, A–C).

To confirm that the mutant (C307A) lost the site-specific SNO while it kept its transcriptional activity, we compared the mRNA levels of the CYP drug-metabolizing enzymes (DMEs) *Cyp3a11*, *Cyp1a2*, and *Cyp2e1* genes in the mouse livers replenished with PXR-WT or the mutant PXR. As shown in Supplemental Figure 12A, the expression levels of these genes were not significantly different at the time before APAP treatment. We further isolated hepatocytes from the *PXR^–/–^* mice and replenished in vitro with PXR-WT or the mutant PXR. Gene expression level of *Cyp3a11*, an endogenous PXR target gene, was not different in the hepatocytes expressing PXR-WT or those expressing the mutant PXR either at the basal level or upon stimulation by the PXR agonist SR12813 (Supplemental Figure 12B). In order to examine the effect of the mutant on human DME, we used a luciferase reporter that was driven by the PXRE from the human *CYP3A4* gene. As shown in Supplemental Figure 12C, neither basal nor SR12813-induced transactivational capacity was significantly different between the PXR-WT and the mutant-PXR–replenished mouse hepatocytes.

**Figure 6:**
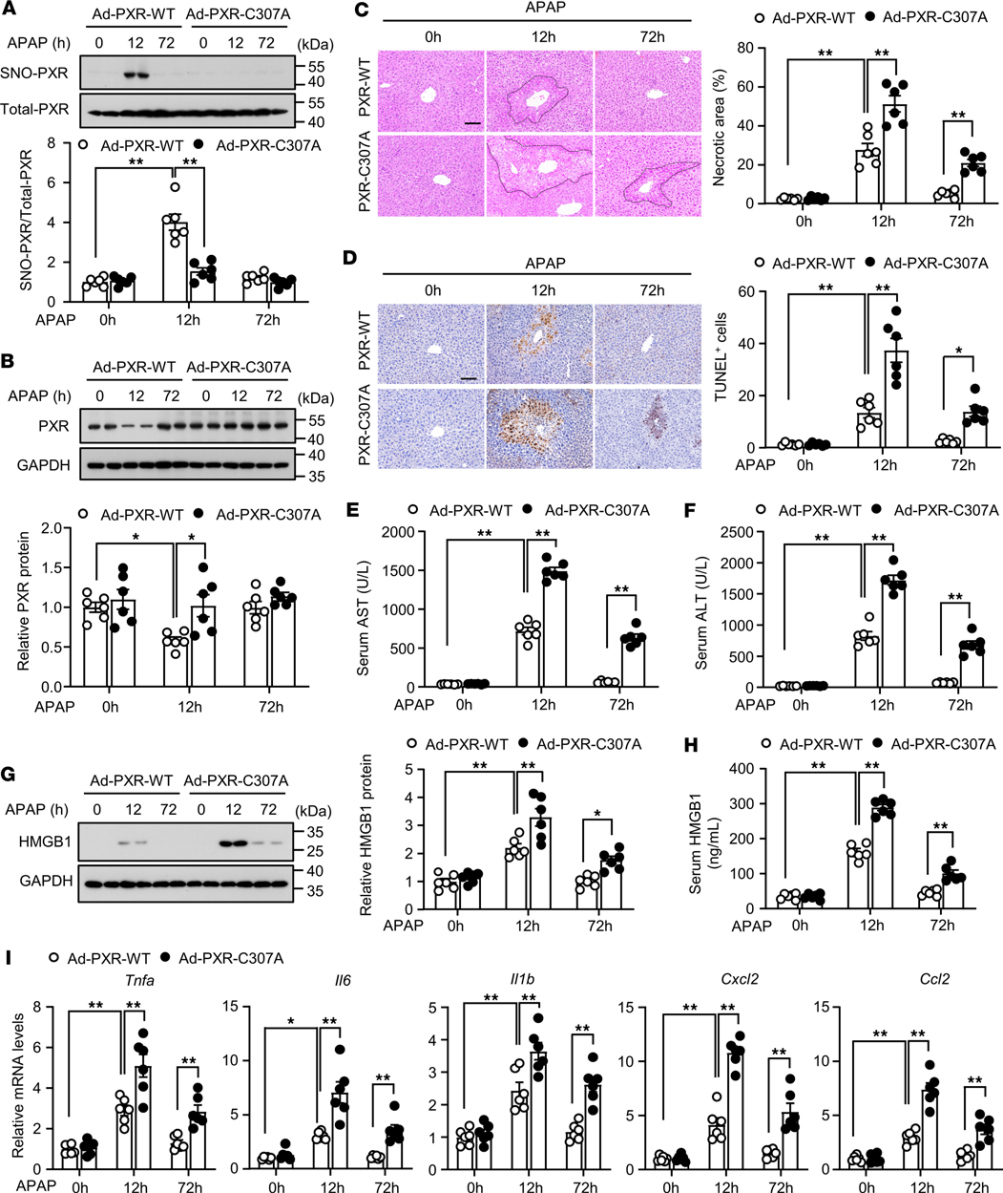
Liver replenishment with the SNO-mutant PXR exacerbated acetaminophen-induced hepatotoxicity in *PXR*-null mice. (**A**) *PXR^–/–^* mice were injected with Ad–PXR-WT or Ad–PXR-C307A. Forty-eight hours later, mice were overdosed with APAP (300 mg/kg) for 12 hours and 72 hours. S-nitrosylated PXR levels in liver tissues were analyzed (*n* = 6). (**B**) PXR protein levels in liver tissues were analyzed (*n* = 6). (**C** and **D**) Mouse liver sections were used for H&E (**C**) and TUNEL (**D**) staining (*n* = 6). Scale bars: 100 μm. (**E** and **F**) Serum AST (**E**) and ALT (**F**) levels in treated mice (*n* = 6). (**G**) HMGB1 protein levels in liver tissues were analyzed (*n* = 6). (**H**) Serum HMGB1 levels in treated mice (*n* = 6). (**I**) The mRNA levels of *Tnfa*, *Il6*, *Il1b*, *Cxcl2*, and *Ccl2* in liver tissues (*n* = 6). All data were expressed as mean ± SEM. Statistical analysis was performed using 1-way ANOVA followed by Tukey’s multiple-comparison test; *P < 0.05, **P < 0.01.

The *PXR^–/–^* mice with hepatic replenishment of PXR-WT or the PXR-C307A were overdosed with APAP (300 mg/kg) and examined at 12 and 72 hours. Compared with the PXR-WT–replenished mice, the PXR-C307A–replenished mice failed to elicit SNO-PXR and degradation upon APAP treatment (Figure 6, A and B). As shown in Figure 6, C–F, compared with the PXR-WT–replenished mice, the PXR-C307A–replenished mice displayed significantly exacerbated hepatocyte death and the serum markers of liver injury with a delayed recovery.

High mobility group box 1 (HMGB1), a chromatin-associated protein and DNA chaperone, plays a critical role in amplifying proinflammatory response in DILI (29). APAP stimulates the expression of HMGB1 and triggers its release from necrotic hepatocytes (30). HMGB1 is passively released from the nucleus and produces related inflammatory factors (31). After APAP treatment, HMGB1 levels in the liver and circulation were further elevated in the PXR-C307A–replenished mice compared with the PXR-WT–replenished mice (Figure 6, G and H). Consequently, the mRNA levels of proinflammatory cytokines, including *Tnfa*, *Il6*, *Il1b*, C-X-C motif chemokine ligand 2 (*Cxcl2*), and chemokine (C-C motif) ligand 2 (*Ccl2*), were further enhanced in the PXR-C307A–replenished livers (Figure 6I). Taken together, our results indicate that SNO-PXR served as a protective mechanism against excessive liver damage.

### Liver replenishment with the SNO-mutant PXR exacerbated APAP-induced reactive metabolites.

**Figure 7:**
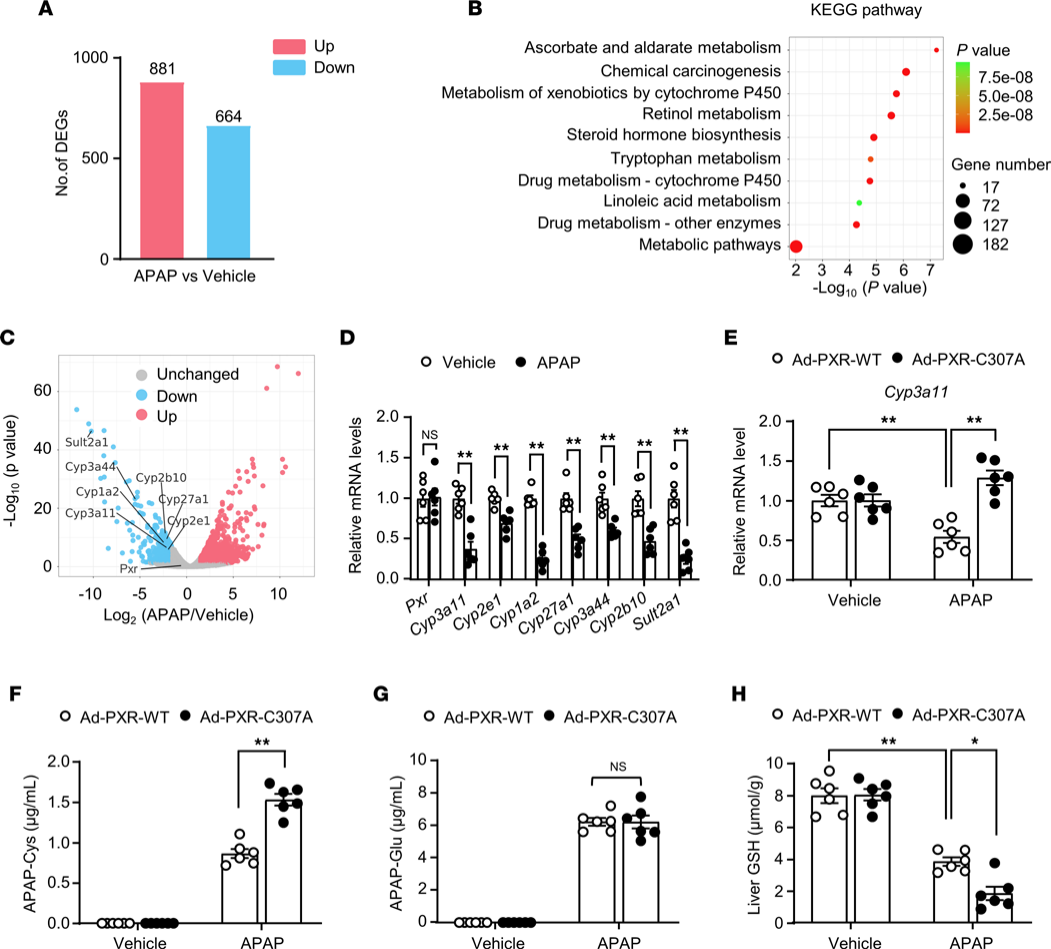
Liver replenishment with the SNO-mutant PXR exacerbated acetaminophen -induced reactive metabolites. (**A**) Summary of DEGs in the livers of APAP overdosed mice (300 mg/kg, 12 hours) compared with the vehicle group. (**B**) Bubble chart shows the KEGG pathway analysis of DEGs. The *x* axis indicates the fold enrichment, and the *y* axis shows the most enriched KEGG pathways. The size of bubble presents genes number and the color of bubble presents the *P* value. (**C**) Volcano plots of the DEGs between APAP group and vehicle group. The *x* axis indicates the fold change (log scaled), and the *y* axis shows the *P* values (log scaled). Each symbol represents a different gene (*P* value and fold change threshold). *P* < 0.05 is considered as statistically significant, and fold change ≥ 1.5 is set as the threshold. *Pxr* and several drug-metabolizing enzymes are labeled. (**D**) The mRNA levels of *Pxr*, *Cyp3a11*, *Cyp2e1*, *Cyp1a2*, *Cyp27a1*, *Cyp3a44*, *Cyp2b10*, and *Sult2a1* were assessed with qPCR (*n* = 6). (**E**) The mRNA levels of *Cyp3a11* were assessed in the livers of PXR-WT–replenished and PXR-C307A–replenished *PXR^–/–^* mice subjected to APAP overdose (300 mg/kg) for 12 hours (*n* = 6). (**F** and **G**) Serum levels of APAP-Cys (**F**) and APAP-Glu (**G**) were detected by using LC-MS/MS (*n* = 6). (**H**) Liver GSH levels in treated mice (*n* = 6). All data were expressed as mean ± SEM. Statistical analysis was performed using 1-way ANOVA followed by Tukey’s multiple-comparison test (**E**–**H**) and 2-tailed Student’s *t* test (**D**); *P < 0.05, **P < 0.01.

Next, we investigated the mechanism by which SNO-PXR protects against liver injury induced by APAP. We performed bulk RNA-Seq in the liver tissue. In total, 1,545 genes were differentially expressed (fold change ≥ 1.5, *P* < 0.05) between the APAP and the control groups, with 881 genes being upregulated and 664 downregulated (Figure 7A). The Kyoto Encyclopedia of Genes and Genomes (KEGG) pathway analysis revealed that the differentially expressed genes (DEGs) were primarily enriched in the pathways involved in the metabolisms of xenobiotics and drug (Figure 7B). Among the DEGs, APAP did not affect the *Pxr* but decreased the expressions of several PXR target genes including *Cyp3a11*, *Cyp27a1*, and *Cyp3a44* (Figure 7C). The high-throughput results were validated by using qPCR (Figure 7D).

Among the PXR target genes, *Cyp3a11* plays an important role in the production of the reactive metabolite NAPQI (32). We compared the hepatic mRNA levels of *Cyp3a11* between the PXR-WT–replenished and PXR-C307A–replenished *PXR^–/–^* mice after APAP overdose. As shown in Figure 7E, the expression level of *Cyp3a11* was significantly increased with PXR-C307A replenishment. We further analyzed APAP metabolites in the mouse serum samples. After APAP overdose, PXR-C307A mice had a significantly increased level of APAP-cysteine (APAP-Cys), which is a protein adduct formed by unstable NAPQI by covalent binding to the cysteine residues (33) (Figure 7F). However, the level of APAP-glucuronide (APAP-Glu) was not different (Figure 7G). Consistently, the levels of hepatic GSH were further reduced in the PXR-C307A–replenished *PXR^–/–^* mice (Figure 7H). Taken together, these results indicate that SNO-PXR might ameliorate APAP-induced hepatotoxicity by inhibiting the formation of reactive metabolites.

### Therapeutic administration of GSNOR inhibitor protected mice from AILI.

**Figure 8:**
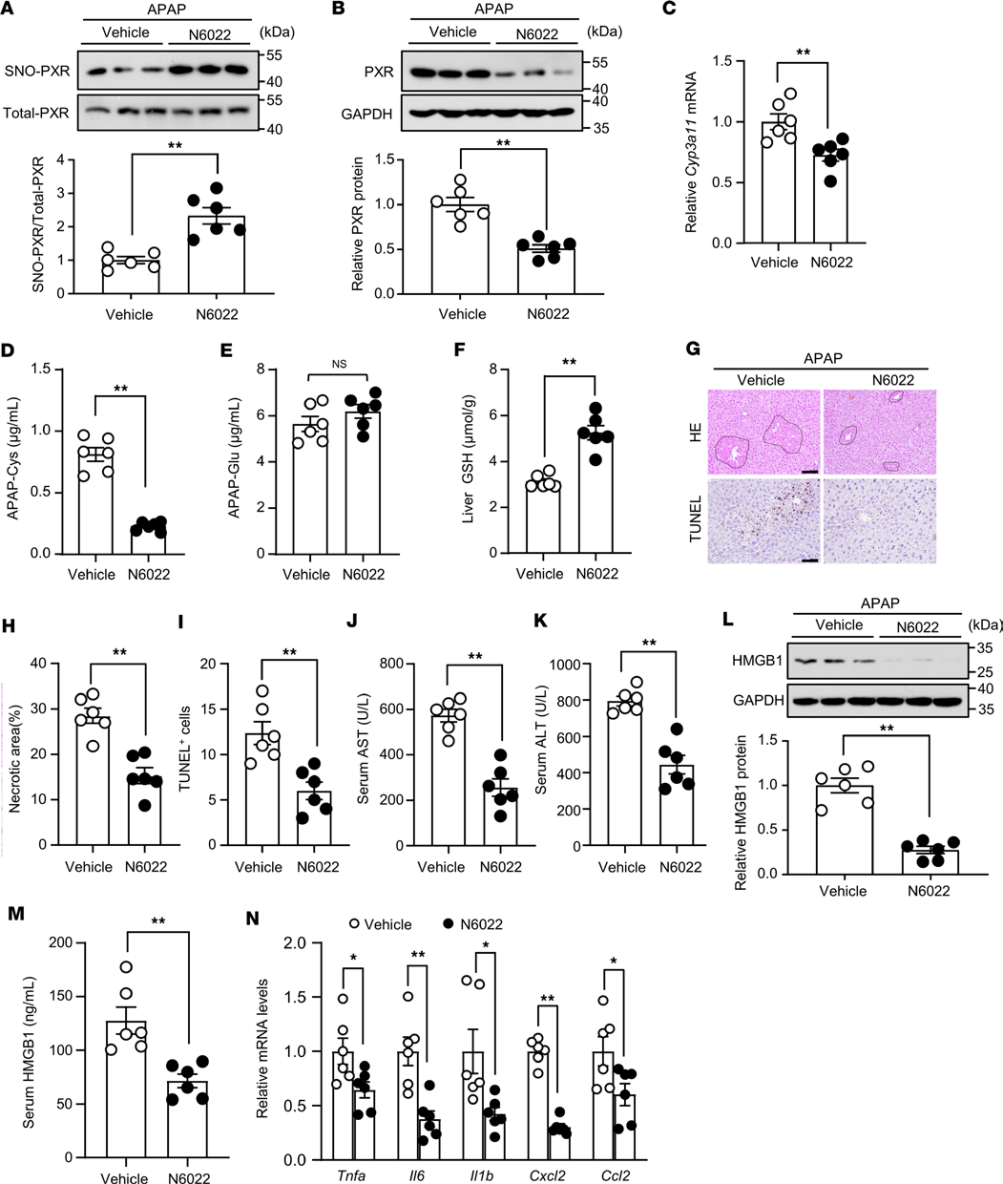
Therapeutic administration of GSNOR inhibitor protected mice from acetaminophen-induced liver injury. C57BL/6J mice were i.p. injected with APAP (300 mg/kg) and, 2 hours later, with N6022 (5 mg/kg). Mice were sacrificed 10 hours later. (**A**) S-nitrosylated PXR levels in liver tissues were analyzed (*n* = 6). (**B**) PXR protein levels in the liver tissues were analyzed (*n* = 6). (**C**) The mRNA level of C*yp3a11* was assessed (*n* = 6). (**D** and **E**) Serum APAP-Cys (**D**) and APAP-Glu (**E**) levels were detected (*n* = 6). (**F**) Liver GSH levels in treated mice (*n* = 6). (**G**) Mouse liver sections were used for H&E (scale bars: 100 μm) and TUNEL (scale bars: 50 μm) staining (*n* = 6). (**H**) Necrotic areas were quantified. (**I**) TUNEL^+^ cells. (**J** and **K**) Serum AST (**J**) and ALT (**K**) levels in treated mice (*n* = 6). (**L**) HMGB1 protein levels in liver tissues were analyzed (*n* = 6). (**M**) Serum HMGB1 levels (*n* = 6). (**N**) The mRNA levels of *Tnfa*, *Il6*, *Il1b*, *Cxcl2,* and *Ccl2* in liver tissues (*n* = 6). All data were expressed as mean ± SEM. Statistical analysis was performed using 2-tailed Student’s *t* test; *P < 0.05, **P < 0.01.

Inhibition of GSNOR results in enhanced levels of SNO (34). C57BL/6J mice were injected with APAP and, 2 hours later, GSNOR inhibitor N6022 (5 mg/kg). As shown in Figure 8, A and B, N6022 increased the SNO of PXR and decreased its protein level in the livers. N6022 significantly decreased the mRNA levels of *Cyp3a11* in the livers and the content of APAP-Cys in the serum samples (Figure 8, C–E). Additionally, N6022 increased hepatic GSH levels (Figure 8F). Histological evaluation showed that N6022 prevented liver necrosis (Figure 8, G–I) and reduced circulating AST and ALT levels (Figure 8, J and K). N6022 downregulated the liver and serum levels of HMGB1 (Figure 8, L and M). Furthermore, N6022 decreased *Tnfa*, *Il6*, *Il1b*, *Cxcl2*, and *Ccl2* mRNA levels in the livers (Figure 8N). These results suggest that the GSNOR inhibitor promoted SNO-PXR and protected the AILI.

## Discussion

In this study, we demonstrated that APAP induced SNO of PXR, which is a posttranslational modification to mitigate the activity of this master regulator of drug metabolism. Nullifying the SNO-PXR exacerbated the liver injury in mice in response to APAP overdose.

Here, we provided both in vitro and in vivo evidence that PXR undergoes SNO in response to APAP or NO donors. This modification was experimentally proven in hepatocytes (HepG2 cell line and mouse primary hepatocytes) and mouse livers (liver extracts with GSNO and liver tissues after APAP overdose) (Figure 1) by using biotin switch assay. SNO was also detected with recombinant human PXR protein by using LC-MS/MS (Supplemental Figure 6). Of functional importance, the SNO negatively regulated PXR activity (Figures 2 and 3). This notion is supported by the following results: (a) GSNO treatment deceased expressions of the PXR target gene as well as the luciferase reporter gene; (b) GSNO decreased the DNA binding of PXR to the regulatory regions of the target genes; (c) GSNO destabilized PXR and decreased its protein level; and (d) disruption of the SNO at cysteine 307 abolished the above-mentioned inhibitory effects on PXR (Figure 4).

The SNO of PXR is of potential importance in the pathogenesis of DILI. This SNO signaling pathway coordinates protective pathways after injury. Our findings show that overdosed APAP led to SNO and degradation of PXR (Figure 5). In addition, the protein level of PXR was decreased at 12 and 24 hours after APAP treatment, whereas SNO levels were increased (Figure 5). The immunofluorescence study also revealed an inverse relationship between the levels of SNO-cysteine and PXR in the livers from the APAP-overdosed mice. Particularly, SNO-cysteine was accumulated at the centrilobular regions where PXR expression was the lowest. Immunoblotting analysis confirmed that APAP triggered SNO of PXR in the mouse livers and subsequently decreased PXR protein levels (Figure 5). Guo et al. (13) previously reported that PCN markedly enhanced AILI in WT but not in PXR-null mice. Further analysis showed that, following PCN treatment, PXR-null mice had lower *Cyp3a11* expression, decreased NAPQI formation, and increased hepatic GSH content compared with WT mice (13). In our present study, abolishing the SNO-PXR resulted in overexpression of *Cyp3a11* and excessive production of APAP-cysteine adducts, leading to exacerbated hepatotoxicity in response to APAP (Figures 6 and 7). Therefore, SNO-PXR may have potential therapeutic value for liver injury. N6022, a first-in-class inhibitor of GSNOR, induced SNO-PXR and protected against APAP-induced hepatotoxicity in mice (Figure 8). In fact, emerging evidence has demonstrated that PXR is a therapeutic target for drug-induced liver injuries (9, 35, 36).

Since SNO is a redox-sensitive and reversible process, this modification may represent a fine-tuning mechanism to adjust PXR activity in response to the tissue milieu and metabolic cues. Our data reveal that SNO-PXR as well as global protein SNO occurred in a time-specific manner in the livers after APAP overdose (Figure 5, G and H). At 72 hours and 96 hours, SNO-PXR returned to the basal level with the concomitant recovery of the cellular and biochemical parameters of liver injury. However, in the PXR-C307A–replenished mice, the recovery was significantly delayed (Figure 6). One of the interpretation is the prolonged inflammatory responses (Figure 6, G–I).

Although our studies focused on the SNO-mediated regulation of the PXR, SNO of other protein substrates may also contribute to the liver protection. This is also suggested by our observation in the ChIP assays where the DNA-binding capacity of the PXR-C307A mutant remained largely preserved under the nitrosative stress (GSNO) but was still lower than that of the PXR-WT (Figure 4F). It is likely explained by the SNO of retinoid X receptor α (RXRα), which is the obligatory dimerization partner of PXR for DNA-binding (37). Cox et al. showed that inhibition of GSNOR resulted in a temporary SNO of nuclear factor erythroid 2-related factor 2 (Nrf2) binding partner Keap1, thereby amplifying the availability of Nrf2 (20). SNO of caspase-8 interrupted mitochondrial apoptotic pathway and protected rat hepatocytes from TNF-α–induced cell death (38). In addition, SNO has been also considered to be protective in the ischemic-reperfusion injury in the hearts (39). In contrast, detrimental roles of SNO have also been described. Yang et al. demonstrated that iNOS-mediated SNO of IRE1α, a key sensor and regulator safe-guarding the protein quality and endoplasmic reticulum (ER) homeostasis, was implicated in the ER dysfunction and the development of obesity (40). GSNOR deficiency in the liver leads to SNO of lysosomal proteins and impaired mitophagy, contributing to obesity and liver insulin resistance (41). Thus, it is conceivable that a role of protein SNO in DILI must be interpreted in a substrate- and context-specific scenario.

PXR regulates drug metabolism in a highly species-specific manner (42). The cysteine residue undergoing SNO in vitro and in vivo (C307 in human PXR) is evolutionarily conserved from zebrafish to humans. Relevance of the SNO-PXR to the human DILI was supported since APAP induced this modification in both mouse and human hepatocytes. Importantly, replenishing the *PXR^–/–^* mouse livers with adenoviruses expressing WT or the SNO-mutant C307A demonstrated that SNO-mediated antagonism of PXR may be an intrinsic mechanism to prevent liver injury due to uncontrollable activation of this xenobiotic nuclear receptor. Our results are in agreement with the previous findings that the gene deficiency in GSNOR led to increased SNO and ameliorated AILI in zebrafish and mice (20), and our results with the C307A mutant suggest that SNO-PXR could be a primary mediator of such effects.

PXR could undergo ubiquitination by RANBP2-type and C3HC4-type zinc finger–containing 1 (RBCK1) (43), ubiquitin protein ligase E3 component N-recognin 5 (UBR5) (44), and tripartite motif containing 21 (TRIM21) (45). The ubiquitinylation led to the proteasome-mediated degradation of PXR. In this study, GSNO and/or APAP caused SNO and concomitant ubiquitination with ensuing degradation in hepatocytes and the liver. The SNO is thought to drive the interplay of this posttranslational modification because nullifying C307 abrogated the ubiquitination and restabilized PXR protein upon the exposure to NO donors and/or APAP. Notably, other forms of posttranslational modifications, including phosphorylation (46), acetylation (47), and SUMOylation (48, 49), have also been shown to either negatively or positively regulate the PXR activity. Poly(ADP-ribosyl)ation of PXR by PARP1 was found to contribute to the AILI in mice (50). The interactions among SNO and these diverse forms of posttranslational modifications warrant in-depth investigation.

In summary, we herein uncovered that SNO PXR plays an important role in APAP-induced hepatotoxicity. Pharmacological targeting of this NO-mediated modification may represent a strategy to manage liver diseases related to drug uses.

## Methods

### Reagents.

MG132, CHX, CQ, sodium ascorbate, methyl-methanethiosulfonate (MMTS), biotin-maleimide, dithiothreitol (DTT), GSNO, streptavidin-agarose, neocuproine, dexamethasone, insulin, HEPES, EGTA, collagenase I, and NaHCO_3_ were from Sigma-Aldrich. APAP was from MedChemExpress. N6022 was from Selleckchem. DETA-NONOate and isopropyl β-D-1-thiogalactopyranoside (IPTG) were from Abcam. APAP-Cys and APAP-Glu were from Toronto Research Chemicals.

### Animals.

Male C57BL/6J, *GSNOR^–/–^*, and *PXR^–/–^* mice (aged 8–10 weeks, weighing 20–25 g) were used in this study. C57BL/6J mice were from Charles River Laboratories. *GSNOR^–/–^* mice were provided by Limin Liu (UCSF, San Francisco, California, USA). *PXR^–/–^* mice were in the C57BL/6J background and generated by deleting Exon 5 with CRISPR/Cas9 system. The genotypes were confirmed by sequencing PCR products of mouse genomic DNA with the primers shown in Supplemental Table 1. All colonies were bred and housed in the M.I.C.E Caging System (Animal Care System) under specific pathogen–free conditions.

### Mouse model of AILI.

After fasting for 12 hours, mice were i.p. injected with freshly made APAP solution (300 mg/kg) or saline and subjected to subsequent analyses at various time points. N6022 was first dissolved in DMSO and diluted with warm PBS before i.p. injection. Blood was collected under isoflurane anesthesia by retro-orbital bleeding. The livers were collected for protein extraction or for histopathological and immunohistological studies.

### Cell culture.

HepG2 and the transformed human embryonic kidney cell line HEK293 were obtained from American Type Culture Collection and maintained in DMEM with 10% FBS (Invitrogen).

Primary mouse hepatocytes were isolated and cultured in RPMI 1640 medium with 10% FBS, insulin (2.5 nM), and dexamethasone (1 nM) (51, 52). Briefly, mouse livers underwent a 2-step collagenase perfusion through the portal vein with Solution 1 (2 mM EGTA, 20 mM HEPES, and 10 mM NaHCO_3_) and Solution 2 (0.05% collagenase I). The livers were minced. The hepatocytes dispersed in culture medium were filtered through a nylon mesh filter and seeded at a density of 1 × 10^7^ cells/10 cm dish on collagen-coated plates (Sigma-Aldrich).

### Detection of SNO-PXR.

SNO of PXR was detected using the IBP method as described previously (18). Briefly, lysed cells or liver tissues were incubated in 20 mM MMTS supplemented with 2.5% sodium dodecyl sulfate (SDS) to block free thiols (50°C, 1 hour). After precipitation with cold acetone, the pellet was resuspended in a 10 mM ascorbate buffer containing 0.2 mM biotin-maleimide for labeling the reduced S-nitrosothiols (37°C, 1 hour). The efficiency of free thiol blocking was examined by performing a negative control without ascorbate. The proteins were boiled in a 200 mM DTT buffer to reduce potential intermolecular disulfide bonds (95°C, 15 minutes). The biotinylated proteins were pulled down using streptavidin-agarose beads and washed with a buffer containing 100 mM NaCl and 2.5% SDS before being denatured in loading buffer. SNO-PXR was analyzed by Western blotting.

### Plasmids.

The full-length human *PXR* gene (NM_003889) was subcloned into pcDNA3.0 (HA tagged) to generate HA-PXR WT expression plasmid. PXR mutants Cys182 (HA-PXR-C182A) and Cys307 (HA-PXR-C307A) were made by using QuikChange Lightning Site-Directed Mutagenesis Kit (Agilent Technologies) and confirmed by DNA sequencing. PXRE-luciferase reporter plasmid (pCYP3A4 XREM –362/+53) contains a PXR enhancer from the 5′-flanking region (–362/+53) of the human CYP3A4 gene and was described previously (53).

### Recombinant adenoviruses and infection.

Recombinant Ad–PXR-WT and PXR-C307A were generated using AdEasy Adenoviral Vector System (Agilent) according to manufactures instruction. Purified Ad–PXR-WT or Ad–PXR-C307A was injected via tail vein into *PXR^–/–^* mice at a dose of 1 × 10^10^ plaque-forming units (PFU). After a 48-hour infection, mice were i.p. injected with APAP (300 mg/kg). Mice were euthanized in a carbon dioxide chamber at 12 hours for collection of serum and liver tissues (*n* = 6/group).

HepG2 cells were infected with Ad-PXR or Ad-GFP (as control vector) at a multiplicity of infection (MOI) of 50 for 2 hours (54). HepG2 cells were also coinfected with Ad–VP-PXR, together with Ad-tTA, encoding a tetracycline-responsive transactivator (a tet-off system) (55). Cells maintained in the presence of tetracycline (1 μg/mL) were used as a control.

### Protein extraction and Western blotting.

Cells and tissues were homogenized with ice-cold RIPA lysis buffer to extract whole protein. Cytoplasmic and nuclear fractions were extracted using hypotonic lysis buffer (10 mM Tris-HCl [pH 7.5], 1.5 mM MgCl_2_, 10 mM KCl, 0.5% [v/v] NP-40) and hypertonic buffer (20 mM Tris-HCl [pH 7.5], 1.5 mM MgCl_2_, 420 mM NaCl, 10% [v/v] glycerol), respectively. Equal amounts of protein were resolved on SDS–polyacrylamide gel electrophoresis (SDS-PAGE) and transferred to polyvinylidene fluoride (PVDF) membrane. Primary and secondary antibodies used are detailed in the Supplemental Table 2. The enhanced chemiluminescence system (ECL) (MilliporeSigma) was used to detect the signal. Densitometric analyses of Western blots were performed using ImageJ software (NIH).

### Ubiquitination assay.

Cells were lysed in ice-cold nondenaturing lysis buffer (50 mM Tris-HCl [pH 7.5], 0.5% NP-40, 120 mM NaCl) supplemented with protease inhibitor cocktail (Roche). Cell lysates were precleared and immunoprecipitated with a mouse monoclonal antibody against PXR and protein G Dynabeads (Invitrogen). The immunoprecipitates were washed with NTEN buffer (20 mM Tris-HCl [pH 8.0], 100 mM NaCl, 1 mM EDTA, 0.5% NP-40), denatured in loading buffer, and subjected to Western blotting with PXR and ubiquitin antibodies.

### qPCR.

Total RNA was extracted by using TRIzol reagent and was reverse transcribed with the iScript RT Supermix containing Moloney murine leukemia virus reverse transcriptase (M-MLV) and oligo (dT) primer mix (Bio-Rad). PCR reactions were performed with the use of specific primers on ABI 7500 real-time PCR system (Applied Biosystems) with SYBR green ﬂuorescence (Promega). Gene expression levels were quantified by using the 2^–ΔΔCt^ method, normalized to GAPDH and expressed as fold induction. Primer sequences are detailed in Supplemental Table 3.

### Immunofluorescence.

Cells were fixed with 4% paraformaldehyde (PFA), blocked with 5% BSA containing 0.3% Triton X-100, and then incubated with the primary rabbit polyclonal antibody against PXR at 4°C overnight. After being washed with PBS, sections were incubated with Alexa Fluor 488–conjugated goat anti–rabbit IgG antibody at 37°C for 1 hour, cells were counterstained with DAPI. Primary and secondary antibodies used are detailed in the Supplemental Table 2.

Mouse liver cryosections were permeabilized with 0.1% Triton X-100, blocked with 3% BSA, and then incubated with primary mouse monoclonal antibody against SNO-Cysteine or the PXR antibody. The sections were incubated with Alexa Fluor 555–conjugated goat anti-mouse or Alexa Fluor 488–conjugated goat anti-rabbit secondary antibodies. Primary and secondary antibodies used are detailed in the Supplemental Table 2. Fluorescence signal was observed using a Nikon E-C2 confocal laser scanning microscope and analyzed using NIS-Elements Advanced Research software (Nikon).

### Luciferase reporter assay.

HEK293 cells were transfected with PXR-WT, PXR-C182A, or PXR-C307A expression plasmids with the PXRE-luciferase reporter plasmid. A β-galactosidase (β-gal) plasmid was cotransfected. Luciferase assays were performed with the use of a luciferase reporter assay system (Promega) according to the manufacturer’s instructions. β-Gal activity was measured to normalize the transfection efficiency.

### Histopathology and IHC analysis.

Paraffin-embedded liver samples were sectioned, dewaxed, and hydrated. Sections were stained with H&E for histopathological evaluation. The quantification of the necrotic area was determined by ImageJ software. Programmed cell death was examined by using ApopTag Peroxidase In Situ Apoptosis Detection Kit (Sigma-Aldrich). TUNEL^+^ cells were counted in 6 randomly selected microscopic fields per section and expressed as percentages of the total number of hepatocytes.

### Biochemical analysis.

Serum AST and ALT were measured using activity colorimetric assay kits (Abcam). Serum HMGB1 levels were determined by using ELISA kit (BioVision). Liver GSH measurements were performed using GSH assay kit (Sigma-Aldrich).

### Expression and purification of recombinant PXR protein.

Human PXR was subcloned into pET-28a (+) vector to produce recombinant protein in *E. coli*. Briefly, BL21 cells were transfected with His-PXR plasmid; the expression was induced by IPTG (0.2 mM, 16°C, 18 hours). Cells were harvested, sonicated, and purified with Ni-NTA agarose beads (Qiagen). The recombinant protein was collected with elution buffer.

### MS determination of S-nitrosylated sites.

Purified His-PXR protein (200 μg) was incubated with GSNO (0.5 mM) for 30 minutes at room temperature. Free thiols were blocked by MMTS, and S-nitrosylated proteins were labeled by biotin-maleimide. Then, the biotinylated proteins were immunoprecipitated with avidin-coupled agarose beads and trypsinized. SNO of the recombinant PXR was detected using LC-MS/MS analysis.

### ChIP assay.

HepG2 cells were cross-linked with 1% formaldehyde for 10 minutes, harvested, and sonicated (25 W, 15 seconds ×3). A mouse monoclonal antibody against PXR (see Supplemental Table 2 for detailed information) or mouse IgG_1_ as a control was used to immunoprecipitate the sheared chromatin. After incubation with protein A/G agarose beads, the immunocomplexes were washed and eluted. DNA was purified and amplified with specific primers (Supplemental Table 4) flanking the known PXRE motifs in the regulatory regions of the human *CYP3A4* and *UGT1A1* genes. Relative DNA binding was expressed as fold enrichment above those amplified from the IgG-precipitated DNA.

### RNA-Seq analysis.

Total RNA was extracted from the livers of APAP-treated or control mice by using TRIzol reagent (Invitrogen). RNA purity and quantification were performed by using NanoDrop 2000 spectrophotometer (Thermo Fisher Scientific). The libraries were constructed and sequenced on an Illumina NovaSeq 6000 platform. FPKM of each gene was calculated using Stringtie, and the read counts of each gene were obtained by HISAT2. Differential expression analysis was performed using the edgeR. *P* < 0.05 was set as the threshold for significantly differential expression. Hierarchical cluster analysis of DEGs was performed to demonstrate the expression pattern of genes. KEGG pathway enrichment analysis of DEGs was performed using R based on the hypergeometric distribution. The transcriptome sequencing and analysis were conducted by SHBIO.

### APAP metabolite analysis.

Blood samples (0.5 mL) were centrifuged (3,000*g*, 4°C, 10 minutes), and the serum samples were snap frozen in liquid nitrogen and stored at –80°C until analysis. APAP-Glu and APAP-Cys were analyzed as previously described (56, 57). Briefly, samples were added at twice the volume of methanol/acetonitrile (1:1, v/v). The mixtures were vortexed for 2 minutes and centrifuged twice at 13,000*g* for 10 minutes at 4°C. Supernatants were injected into LC-MS/MS (Shimadzu), operated in the negative ion electrospray mode with selective ion monitoring. A WondaCract ODS-2 column (5 μm, 150 × 4.6 mm) was used for chromatographic separation. Methanol/acetonitrile (1:1, v/v) and 0.1% formic acid and water (55:45) were used as the mobile phase delivered at a flow rate of 0.5 mL/min. The following mass spectrometric conditions were used: curtain gas, 25 psi; temperature, 500°C; GS1 (nebulizer gas), 40 psi; GS2 (heating gas), 30 psi; collision energy, 35 eV (for glucuronide) or 24 eV (for cysteine); and declustering potential, 34 V (for glucuronide) or 35 V (for cysteine). The multiple reaction monitoring (MRM) conditions used for APAP-Glu and APAP-Cys were *m/z* (mass-to-charge ratio) 326→150 and *m/z* 268.9→182 (production spectra). Standard curves corresponded to peak area ratios of each analyte using weighted linear least squares regression for APAP-Glu and APAP-Cys; the linearity coefficients of determination (*R*^2^) were 0.99994 and 0.9987, respectively. Concentrations of metabolites were measured by comparing the peak area ratios after the unknown samples to the standard curve for each metabolite. The pharmacokinetic parameters were estimated by using the WinNonlin software from Pharsight.

### Plasmids and siRNA transfection.

Transient transfection was performed using Lipofectamine 3000 (for plasmids) or Lipofectamine RNAiMAX (for siRNA) according to the manufacturer’s instructions (Invitrogen). The siRNA sequences targeting mouse *Inos* were shown in Supplemental Table 5. The siRNA with scrambled sequence was used as a negative control (NC siRNA).

### Statistics.

All data are reported as mean ± SEM, and statistical analyses were performed in Prism 8 (GraphPad Software). All experiments had at least 3 individual biological repeats. Comparisons between 2 groups were analyzed using 2-tailed, unpaired Student’s *t* test. Comparisons between 3 or more groups were performed using 1-way or 2-way ANOVA followed by Tukey’s or Bonferroni’s test. *P* values less than 0.05 were considered statistically significant.

### Study approval.

The study was approved by the IACUC of Animals of the Dalian Medical University (AEE18066) and Xi’an Jiaotong University (2022-1384).

### Data availability.

The data that support the findings of this study are available within the article and its supplemental tables and figures. All data in the manuscript and supplemental material presented in graphs are provided in the Supporting Data Values file. The raw RNA-Seq data have been deposited in the NCBI Gene Expression Omnibus database (GEO GSE243679). Any additional information required to reanalyze the data reported in this paper is available from the corresponding author upon request.

## Author contributions

QC, TJ, XX, TL, QY, JL, and CC performed the experiments and analyzed the data; HW, YC, QL, LQ, and BL performed the experiments; QC drafted the paper; and QC, LX, and NW designed the study, analyzed the results, and revised the manuscript. All authors read and approved the manuscript.

## Supplementary Material

Supplemental data

Supporting data values

## Version 1 11/30/2023

In-Press Preview

## Version 2 01/23/2024

Electronic publication
