# Supplementary material for: S-nitrosylation attenuates pregnane X receptor hyperactivity and acetaminophen-induced liver injury
Source: JCI Insight. 2024 Jan 23;9(2):e172632. doi: 10.1172/jci.insight.172632 (PMC10906221; doi:10.1172/jci.insight.172632)
Supplement: Supplemental data [file jciinsight-9-172632-s009.pdf]

S-nitrosylation Attenuates Pregnane X Receptor Hyperactivity and Acetaminophen-induced Liver Injury

Qi Cui[1,2], Tingting Jiang[1,2], Xinya Xie[2], Haodong Wang[3], Lei Qian[1], Yanyan Cheng[1], Qiang Li[4], Tingxu Lu[2], Qinyu Yao[2], Jia Liu[2], Baochang Lai[2], Chang Chen[5], Lei Xiao[2*] and Nanping Wang[3*]

[1]Advanced Institute for Medical Sciences, Dalian Medical University, Dalian, 116044, China

[2]School of Basic Medical Sciences, Xi'an Jiaotong University, Xi'an, 710061, China

[3]East China Normal University Health Science Center, Shanghai, 200241, China

[4]School of Public Health, Xi'an Jiaotong University, Xi'an, 710061, China

[5]National Laboratory of Biomacromolecules, Center for Excellence in Biomacromolecules, Institute of Biophysics, Chinese Academy of Sciences, Beijing, 100101, China

[*]Correspondence:

Lei Xiao, PhD,

Key Laboratory of Environment and Genes Related to Diseases, Xi'an Jiaotong University, Ministry of Education of China; Xi'an, 710061, China

Tel: +86-029-82655186, Fax: +86-029-82655196. E-mail: xiaolei0122@xjtu.edu.cn or

Nanping Wang, MD, PhD,

East China Normal University Health Science Center, Shanghai, 200241, China

Tel: +86-021-62235057, Fax: +86-021-62235057. E-mail: npwang@hsc.ecnu.edu.cn.

Supplemental Figure 1

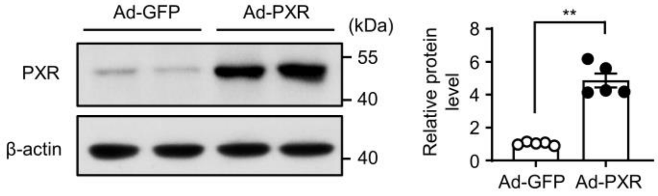

Supplemental Figure 1. Overexpression of PXR in HepG2 cells. HepG2 cells were infected with Ad-PXR or Ad-GFP for 24 h. Total PXR protein level was measured by using western blotting (n=5). All data were expressed as mean ± SEM. Statistical analysis was performed using 2-tailed Student's *t* test; $^{**}P < 0.01$.

Supplemental Figure 2

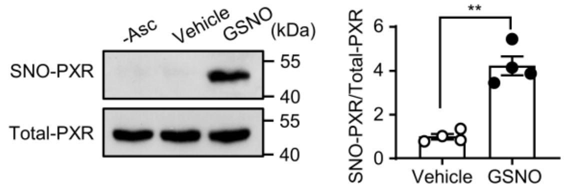

Supplemental Figure 2. PXR was S-nitrosylated in HepG2 cells. HepG2 cells were exposed to GSNO (0.5 mM, 4 h). Cell lysates were subjected to IBP to detect S-nitrosylated PXR level (n=4). –Asc, ascorbate acid (Asc) was omitted to serve as the negative control of biotin-switch assay. Statistical analysis was performed using 2-tailed Student's *t* test; $^{**}P < 0.01$.

Supplemental Figure 3

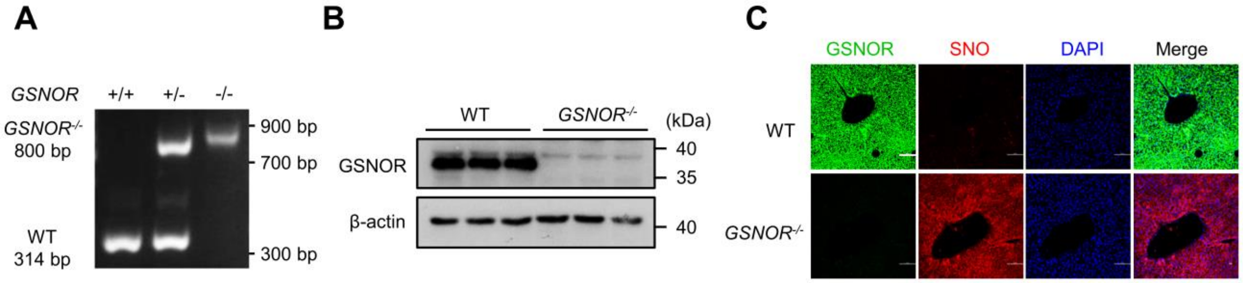

Supplemental Figure 3. ***GSNOR*$^{-/-}$ mice displayed increased protein S-nitrosylation in the liver.** (**A**) PCR genotyping results for *GSNOR*$^{+/+}$ (WT), *GSNOR*$^{+/-}$ and *GSNOR*$^{-/-}$ mice. (**B**) GSNOR protein levels in liver samples from the WT and *GSNOR*$^{-/-}$ mice were detected (n = 3 for each group). (**C**) WT and *GSNOR*$^{-/-}$ mouse liver sections were subjected to immunofluorescence double-stained with primary antibodies against GSNOR and SNO-cysteine (SNO), and followed by the detection with Alexa Fluor 488 (green)-conjugated and Alexa Fluor 555 (red)-conjugated secondary antibodies. The cell nuclei were counterstained with DAPI (n = 3 for each group). Scale bars, 100 µm.

Supplemental Figure 4

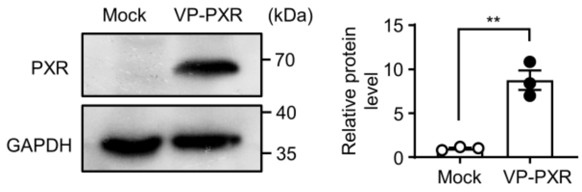

Supplemental Figure 4. Overexpression of Ad-VP-PXR in HepG2 cells. Confluent HepG2 cells were co-infected with Ad-VP-PXR together with Ad-tTA in the presence (Mock) or absence of tetracycline (1 μg/mL). Total PXR protein level was measured (n=3). Statistical analysis was performed using 2-tailed Student’s *t* test; $^{**}P < 0.01$.

Supplemental Figure 5

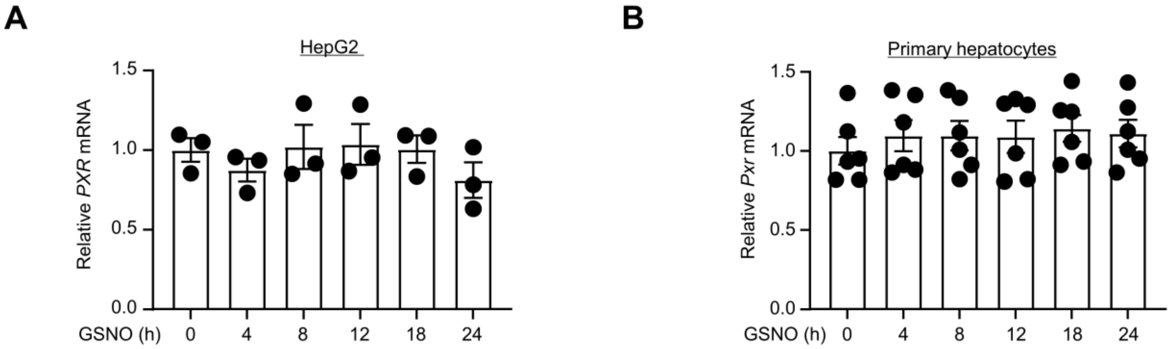

Supplemental Figure 5. GSNO did not affect PXR mRNA level. HepG2 cells (**A**, n=3) or mouse primary hepatocytes (**B**, n=6) were exposed to GSNO for indicated time periods. PXR mRNA levels were assessed. Data were expressed as mean ± SEM. Statistical analysis was performed using 1-way ANOVA followed by Tukey’s multiple comparison test.

Supplemental Figure 6

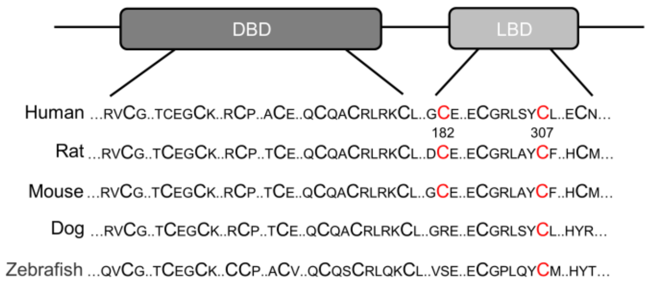

Supplemental Figure 6. Conserved cysteine residues in PXR protein. Sequence alignment of conserved cysteine residues in human, rat, mouse, dog and zebrafish PXR proteins. LBD, ligand-binding domain; DBD, DNA-binding domain.

Supplemental Figure 7

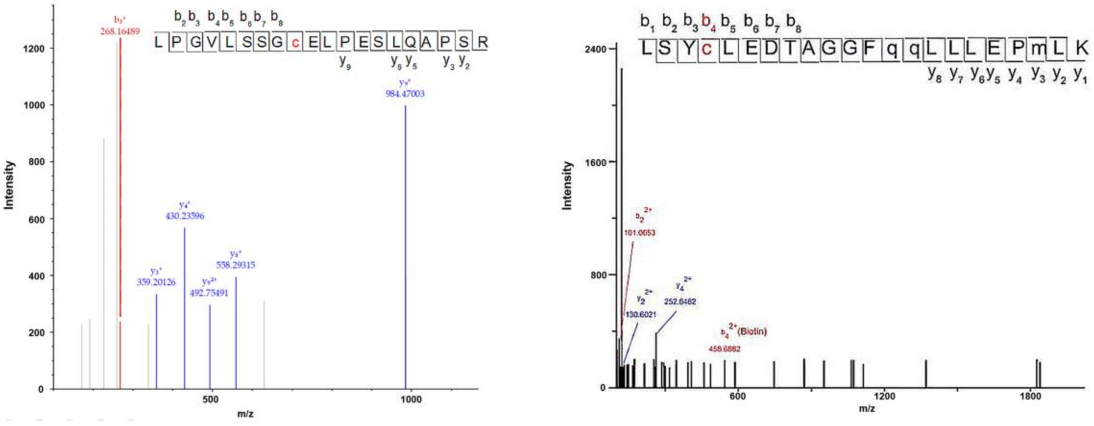

Sequence	Sites	Charge	Calc. MH+ [Da]	ΔM [ppm]
LPGVLSSGCELPESLQAPSR	Cys182	3	2491.2399	3.17
LSYCLEDTAGGFqqLLLEPmLK	Cys307	4	2936.3490	-9.44

Supplemental Figure 7. Identification of S-nitrosylated cysteine residues in PXR. Representative LC-MS/MS spectra of biotin-maleimide labeled (S-nitrosylated) peptides in the recombinant human PXR. Sequence informative fragmentation ions were annotated (red, b-ions; blue, y-ions) on the peptide sequences and summarized with the sites, charge, calculated (Calc.) and measured mass in parts per million (p.p.m.).

Supplemental Figure 8

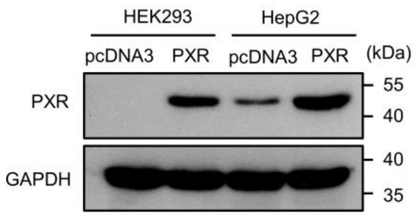

Supplemental Figure 8. Detection of PXR protein in HEK293 cells. HEK293 or HepG2 cells were transfected with HA-PXR (HA-tagged PXR) or pcDNA3.0 as control plasmid. Total PXR protein levels were measured 24 h later using western blotting (n=2 for each group).

Supplemental Figure 9

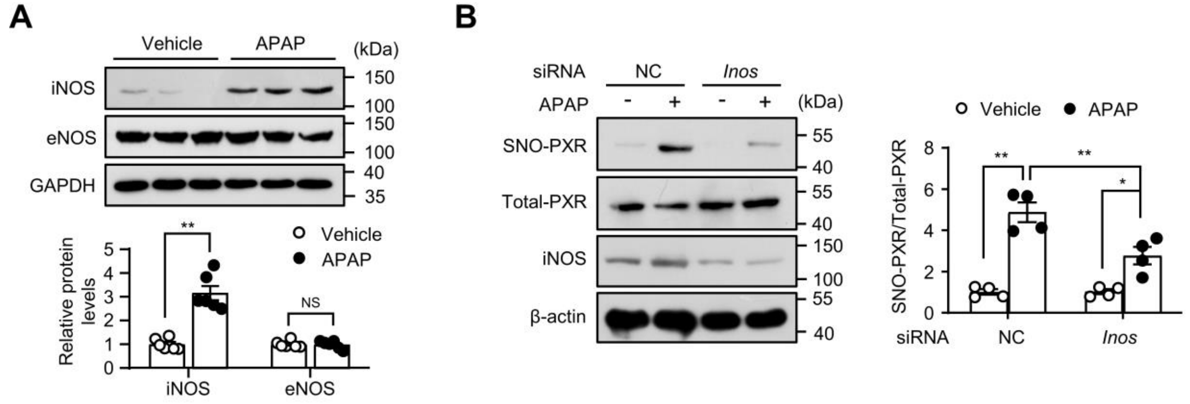

Supplemental Figure 9. (**A**) Western blotting was performed to detect the expressions of iNOS and eNOS in the mouse livers with or without APAP overdose (300 mg/kg, or vehicle for 12 h) (n=6). (**B**) Mouse primary hepatocytes were transfected with double-stranded *Inos* siRNA or scrambled (NC) siRNA. Twenty-four h later, the cells were treated with APAP (5 mM, 12 h) or vehicle control (saline). The protein levels of iNOS and S-nitrosylated PXR were detected (n=4). Data were expressed as mean ± SEM. Statistical analysis was performed using 2-tailed Student's *t* test (**A**) and 1-way ANOVA followed by Tukey's multiple comparison test (**B**); $^{**}P < 0.01$. NS, not significant.

Supplemental Figure 10

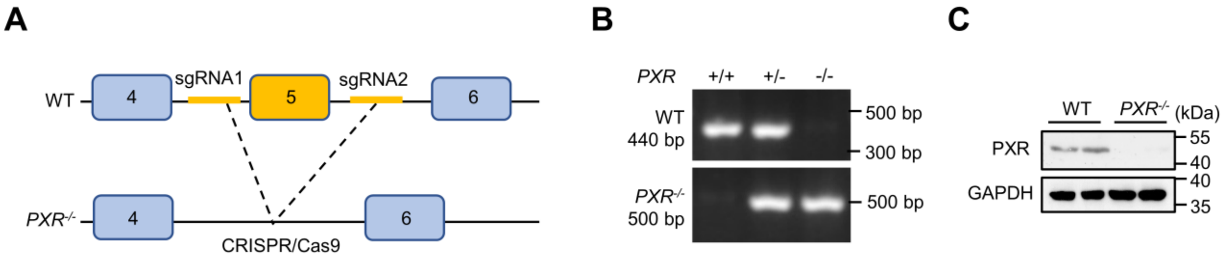

Supplemental Figure 10. Generation of *PXR*$^{-/-}$ mice. (**A**) *PXR*$^{-/-}$ mice were generated by deleting exon 5. (**B**) PCR genotyping results for *PXR*$^{+/+}$ (WT), *PXR*$^{+/-}$ and *PXR*$^{-/-}$ mice. (**C**) Liver PXR protein levels in WT and *PXR*$^{-/-}$ mice (n=6 for each group).

Supplemental Figure 11

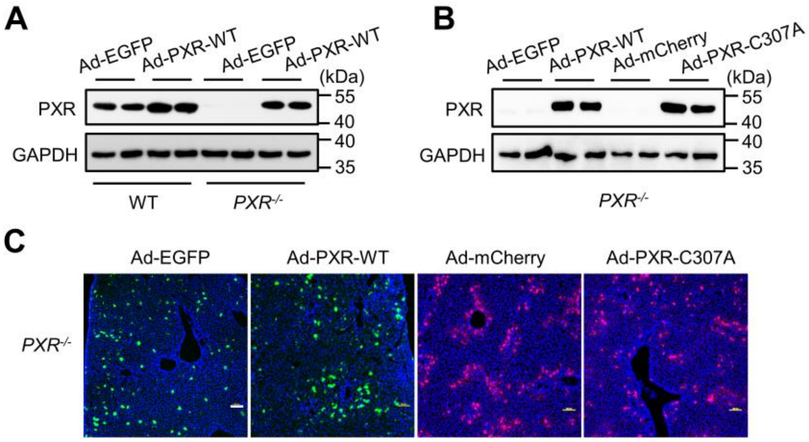

Supplemental Figure 11. Replenishment of PXR-WT or PXR-C307A in *PXR*$^{-/-}$ mice. (**A**) WT or *PXR*$^{-/-}$ mice at 8-10 weeks of age were injected with 100 μL Ad-EGFP or Ad-PXR-WT virus containing 1×10^{10} PFU via tail vein. After 60 h, PXR protein levels were measured (n=2 for each group). (**B**) *PXR*$^{-/-}$ mice were injected with 100 μL Ad-EGFP, Ad-PXR-WT, Ad-mCherry or Ad-PXR-C307A. After 60 h, the efficiency of adenovirus-mediated PXR overexpression was measured (n=2 for each group). (**C**) Fluorescence microscopy of mouse liver tissues was acquired (n=2 for each group). Scale bars, 100 μm.

Supplemental Figure 12

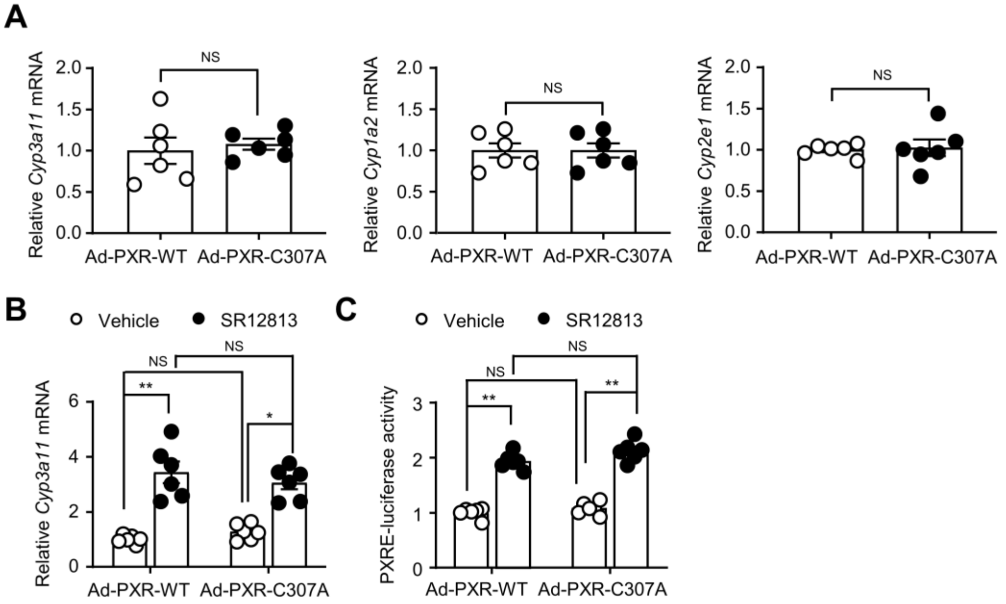

Supplemental Figure 12. (**A**) The mRNA levels of *Cyp3a11, Cyp1a2* and *Cyp2e1* were assessed in the livers 48 h after Ad-PXR-WT or Ad-PXR C307A-mediated replenishment of the *PXR*$^{-/-}$ mice (n=6). (**B**) Primary hepatocytes were isolated from the *PXR*$^{-/-}$ mice and infected with Ad-PXR-WT or Ad-PXR-C307A. Twenty-four h later, cells were treated with or without SR12813 (1 μM, 24 h) (n=6). (**C**) The primary hepatocytes from *PXR*$^{-/-}$ mice were first transfected with PXRE-luciferase receptor and β-gal plasmids and, 6 h later, infected with Ad-PXR-WT or Ad-PXR-C307A. Twenty-four h later, the cells were exposed to SR12813 (1 μM, 24 h) or vehicle. The luciferase activities were measured and normalized to β-gal activity (n=6). All data were expressed as mean ± SEM. Statistical analysis was performed using 2-tailed Student's *t* test (**A**) and 1-way ANOVA followed by Tukey's multiple comparison test (**B** and **C**); $^{*}P$ <0.05, $^{**}P$ <0.01. NS, not significant.

Supplemental tables

Supplemental Table 1. PCR primers used for genotyping

Name	Sequence (5'-3')
GSNOR-WT	Forward TGCCTTCTCGGCTGTGGT
	Reverse GGCCTTTGCGAATTTATCTTTA
GSNOR-KO	Forward TCTTGACGAGTTCTTCTGAGG
	Reverse CTGAAGCAGCTACTCCCACTAC
PXR-WT	Forward TGCTGTAGGGCTGATGTGTG
	Reverse TGCGTGTGTGATTTAGCCCT
PXR-KO	Forward AGGGGTACGGACTTCCTGTT
	Reverse TCTCTTTGGGCACCATTAGG

Supplemental Table 2. Antibody information

**Antibody**	**Company**	**Catalog #**	**Application/Dilution**
β-actin (C-2)	SCBT	sc-8432	WB (1:1000)
eNOS (D9A5L)	CST	32027	WB (1:1000)
Flag-Tag	Sigma-Aldrich	F3165	WB (1:1000)
GAPDH (D16H11)	CST	5174	WB (1:1000)
GSNOR	Proteintech	11051-1-AP	WB (1:1000)
HA-Tag (F-7)	SCBT	sc-7392	WB (1:1000)
Histone (AH3-120)	SCBT	sc-56616	WB (1:1000)
HMGB1(D3E5)	CST	6893	WB (1:1000)
iNOS (H-174)	SCBT	sc-8310	WB (1:1000)
PXR	Abcam	ab192579	IF (1:100)
PXR	Arigobio	ARG43116	WB (1:1000), IF (1:200)
PXR (G-11)	SCBT	sc-48403	IP (1:50)
SNO-Cysteine	Abcam	ab94930	WB (1:1000), IF (1:400)
Ubiquitin	CST	3933	WB (1:1000)
Alexa Fluor 555 anti-rabbit secondary antibody	CST	4413	IF (1:500)
Alexa Fluor 488 anti-rabbit secondary antibody	CST	4412	IF (1:500)
Alexa Fluor 555 anti-mouse secondary antibody	CST	4409	IF (1:500)
Alexa Fluor 488 anti-mouse secondary antibody	CST	4408	IF (1:500)
Anti-rabbit IgG, HRP-linked Antibody	CST	7074	WB (1:5000)
Anti-mouse IgG, HRP-linked Antibody	CST	7076	WB (1:5000)

WB, western blotting; IP, immunoprecipitation; IF, immunofluorescence

Supplemental Table 3. Primers used for qRT-PCR

Name	Sequence (5'-3')	
CYP3A4 (human)	Forward	AATCCTAGCAGTTTGGGAGGCTGA
	Reverse	TGAGATTACAGGCGAGTCCACCAT
SULT1A1 (human)	Forward	CACGTCGTTCAAGGAGATGA
	Reverse	AGGTTTGATTCGCACACTCC
PXR (human)	Forward	AACTCGCAGCCACTGCTAAG
	Reverse	ACCACCAAGCAGTCCAAGAG
GAPDH (human, mouse)	Forward	ACCACAGTCCATGCCATCAC
	Reverse	TCCACCACCCTGTTGCTGTA
Pxr (mouse)	Forward	CACAACTTTCTCCCACTTCAAG
	Reverse	CCTTGAACATGTAGGTTGACAC
Il1b (mouse)	Forward	GTGCAAGTGTCTGAAGCAGC
	Reverse	CAAAGGTTTGGAAGCAGCCC
Il6 (mouse)	Forward	TCCAGTTGCCTTCTTGGGAC
	Reverse	GTACTCCAGAAGACCAGAGG
Tnfa (mouse)	Forward	GGCTGCCCCGACTACGT
	Reverse	ACTTTCTCCTGGTATGAGATAGCAAAT
Cxcl2 (mouse)	Forward	CCAGCCTACTCATTGGGAT
	Reverse	GGGCCTGCTGTTCACAGTT
Ccl2 (mouse)	Forward	TCCAGGTCAGTTAGCCTTGC
	Reverse	CGGTCAAAAAGTTTGCCTTG
Cyp3a11 (mouse)	Forward	ACAGCACTGGTCAGAGCCTGAA
	Reverse	GAGAGCAAACCTCATGCCAAGG
Cyp1a2 (mouse)	Forward	CATCACAAGTGCCCTGTTCAAGC
	Reverse	AATGCTCCAGGTGATGGCTGTG
Cyp2e1 (mouse)	Forward	GGCTGTCAAGGAGGTGCTACT
	Reverse	AAAACCTCCGCACGTCCTTCCA
Cyp27a1 (mouse)	Forward	TCAGGAGACCATCGGCACCTTT
	Reverse	CCAGTCACTTCCTTGTGCAAGG
Cyp3a44 (mouse)	Forward	TGCTCTTCACCATGACCCACAG
	Reverse	CCTCATGCCAATGCAGTTCCTG
Cyp2b10 (mouse)	Forward	AAGGAGAAGTCCAACCAGCA
	Reverse	CTCTGCAACATGGGGGTACT
Sult2a1 (mouse)	Forward	GGAAGGACCACGACTCATAACC
	Reverse	CTCTGGGATTTCTCACGAGATAG

Supplemental Table 4. Primers used for ChIP assay

Gene	Sequence (5'-3')
CYP3A4	Forward TGGTTCATTCCTTTCATTTGAT
	Reverse AGCAGAGGGTCAGCAAGTTC
UGT1A1	Forward GACACTGGATTCTTTGCTTTGAT
	Reverse AGTCCGGGTTTCAGGTTATG

Supplemental Table 5. Sequences of primers for RNA silencing

Gene	Sequence (5'-3')
Scrambled control	Sense CGGGUUGCCCAAAGACGACAA
	Antisense UUGUCGUCUUUGGGCAACCCG
Inos	Sense CCAGUUGUGCAUCGACCUA
	Antisense UAGGUCGAUGCACAACUGG
